# *Mycobacterium smegmatis* NucS-promoted DNA mismatch repair involves limited resection by a 5′-3′ exonuclease and is independent of homologous recombination and NHEJ

**DOI:** 10.1093/nar/gkae895

**Published:** 2024-10-17

**Authors:** Iris V Rivera-Flores, Emily X Wang, Kenan C Murphy

**Affiliations:** Department of Microbiology, University of Massachusetts Chan Medical School, Worcester, MA 01605, USA; Department of Microbiology, University of Massachusetts Chan Medical School, Worcester, MA 01605, USA; Department of Microbiology, University of Massachusetts Chan Medical School, Worcester, MA 01605, USA

## Abstract

The MutSL mismatch repair (MMR) systems in bacteria and eukaryotes correct mismatches made at the replication fork to maintain genome stability. A novel MMR system is represented by the EndoMS/NucS endonuclease from Actinobacterium *Corynebacterium glutamicum*, which recognizes mismatched substrates *in vitro* and creates dsDNA breaks at the mismatch. In this report, a genetic analysis shows that an *M. smegmatis* Δ*nucS* strain could be complemented by expression of wild type NucS protein, but not by one deleted of its last five amino acids, a region predicted to be critical for binding to the β-clamp at the replication fork. Oligo-recombineering was then leveraged to deliver defined mismatches to a defective hygromycin resistance gene on the *M. smegmatis* chromosome. We find that NucS recognizes and repairs G-G, G-T, and T-T mismatches *in vivo*, consistent with the recognition of these same mismatches in *C. glutamicum in vitro*, as well as mutation accumulation studies done in *M. smegmatis*. Finally, an assay that employs an oligo that promotes the generation of two mismatches in close proximity on the chromosome shows that a NucS-promoted cut is processed by a 5′–3′ exonuclease (or 5'-Flap endonuclease) and that NucS-promoted MMR is independent of both homologous recombination and non-homologous end-joining.

## Graphical Abstract

**Graphical Abstract:**
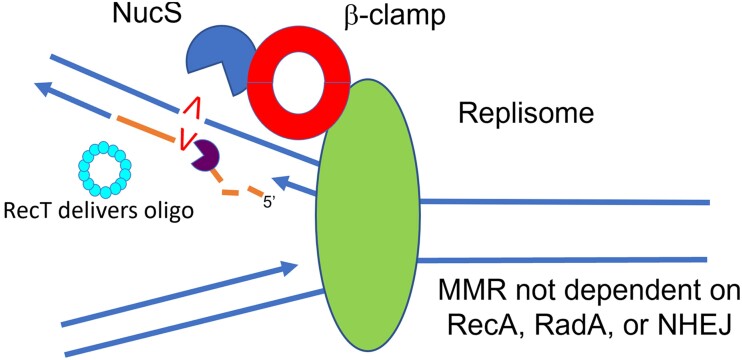

## Introduction

High fidelity of DNA replication in all organisms is of paramount importance to minimize mutation accumulation and maintain genome stability. Most cells correct the misincorporation of the wrong nucleotide during DNA replication by two distinct mechanisms. The first line of defense is provided by the proofreading functions of DNA polymerases that immediately detect and exonucleolytically remove an incorrect nucleotide in the nascent DNA strand (1–3). Should this system fail, however, DNA mismatch repair (MMR) systems recognize mismatched base pairs in the wake of the replication fork, identify which base of the mismatch is the incorrect one, and processes its removal (4,5). Resynthesis of the exposed template and religation to the undamaged preexisting DNA strand completes the repair process. The canonical MMR pathway is represented by the MutSL system, which was first identified in *Escherichia coli* (6–8). The *E. coli* MutS protein recognizes the mismatched bases and together with MutL promotes a platform for nicking of the newly synthesized strand by MutH endonuclease, governed by the hemi-methylation status of DNA. In most bacteria, however, *mutH* is not present and a latent MutL endonuclease is responsible for cutting the replicative strand (for reviews, see (8–11)).

Most archaeal and many actinobacterial genomes (including mycobacteria) are devoid of the canonical MutSL MMR system. It was believed for many years that perhaps these organisms did not possess an MMR system. However, these species do not show higher rates of mutagenesis relative to bacteria that contain MutSL systems, suggesting that DNA replication fidelity in these bacteria relies solely on an efficient polymerase proofreading mechanism, or that an alternative MMR system exists in these species. The first hint of such a repair system came from the studies of Ishino *et al.* (12) that described an endonuclease from the hyperthermophilic archaeon *Pyrococcus furiosus* that specifically recognizes and cuts mismatched dsDNA, which they called EndoMS (endonuclease specific for mismatch-specific DNA). EndoMS is homologous to a protein previously isolated from *Pyrococcus abyssi*, that was identified as a nuclease specific for ssDNA, called NucS (13), which is the name used for this protein hereafter.

Ichino *et al* (12) went on to study the NucS protein from *Thermococcus kodakarensis* (TKO), a more genetically tractable thermophilic archaeon. They found that the TKO NucS specifically binds to and cuts both strands of dsDNA species that contained G–T, G–G and T–T mismatches. Higher concentrations of the enzyme were required to cut dsDNA containing T–C and A–C mismatches, though such substrates failed to bind TKO NucS in an electrophoretic mobility shift assay. DNA substrates containing C–C, A–C and A–A showed no binding or cutting of mismatched substrates.

NucS proteins have no homology to any of the components of the mutSL systems, and thus was the first protein from an archaeon that suggested the existence of an alternative MMR system (12,14). From these studies, it was found that TKO NucS cuts on both sides of the mismatch leaving 5′-P and 3′-OH ends with 5′ protruding overhangs, reminiscent of the action of restriction enzymes. Indeed, the structure of the TKO NucS bound to a 15-mer mismatched-containing dsDNA was solved by Nakae *et al.* (14) showing that NucS protein to be remarkably similar to the structure of type II restriction enzymes, with the N-terminal domain promoting dimerization of NucS and the C-terminal domain binding to regions flanking the mismatched bases. The two bases of the mismatch were flipped out of the helix into binding sites within the protein, not unlike the recognition sequence of the Ecl18kl restriction enzyme (CCNGG), where the central base pair is flipped out in a manner like NucS acting on a mismatch. Unlike restriction enzymes, however, NucS does not have a recognition sequence in that it does not contact any bases at the cut site except for those contained within the mismatch (14). The recognition and cutting of mismatches by TKO NucS highlight the difference between this novel MMR system and that of MutSL, where MutS recognizes the mismatch, but a single-stranded DNA nick is made either by MutH directed to GATC sites on the unmethylated strand of the replication fork (*e.g*. in *E. coli*), or in other bacteria and eukaryotes, by a β-clamp activation of a latent MutL endonuclease that nicks the newly synthesized strand. For recent updates on both MutS and NucS endonuclease function and strand discrimination in MMR, see the following reviews (15,16).

Of particular interest is whether the novel NucS MMR system exhibited in the archaea described above is active as an anti-mutator in Actinobacteria where the gene is present, including the clinically relevant bacterium *Mycobacterium tuberculosis*. The question was addressed by two labs (17,18) that studied actinobacterium *Corynebacterium glutamicum* and found that deletion of the *nucS* homolog (NCgl-1168, hereafter called NucS_Cg_) resulted in a strain with a mutator phenotype. Purification of the NucS_Cg_ protein showed that it bound to DNA containing base pair mismatches, and that cutting of these substrates was activated by an interaction with the β-clamp from *C. glutamicum*, DnaN_Cg_. The mismatch recognition and cutting specificities of NucS_Cg_ were the same as those observed for TKO NucS and were consistent with the mutation spectrum of *C. glutamicum* in mutation accumulation (MA) studies done in the *nucS*-deficient background, i.e. a higher levels of transitions observed relative to wild type (18).

The question of whether NucS from a mycobacterial species could bind mismatched DNA was addressed by Castañeda-Garcia *et al.* (19) who examined thousands of transposon mutants in *Mycobacterium smegmatis* for high rates of spontaneous resistance to rifampicin. They identified MSMEG_4923 as a gene responsible for generating a mutator phenotype in *M. smegmatis*; this gene product has 27% sequence identity with NucS from *P. abyssi* and 73% sequence identity with NucS_Cg_. However, isolation and characterization of the *M. smegmatis* NucS protein showed that while it bound to ssDNA and not dsDNA (like its *P. abyssi* homolog), it did not demonstrate binding activity to mismatch-containing dsDNA substrates *in vitro*. This result contrasts with those from the NucS proteins from *P. furiosus* and *T. kodakarensis in vitro*, raising the question of whether the mycobacterial NucS acts *in vivo* in a similar way these proteins *in vivo*.

We have addressed this question here genetically by examining if *M. smegmatis* NucS requires an interaction with the β-clamp *in vivo*, as was seen previously with the NucS protein from *T. kodakarensis in vitro*. Complementation studies with plasmid-encoded wild type NucS and a derivative containing a C-terminal 5 amino acid truncation suggest an interaction with the *M. smegmatis* β-clamp is required for MMR activity *in vivo*. Secondly, we examined the mismatch specificity of *M. smegmatis* NucS *in vivo* and compared it to the mismatch specificities of *T. kodakarensis* and *C. glutamicum* reported *in vitro*. We used Che9 RecT-promoted oligo recombineering in *M. smegmatis* to directly deliver all possible types of base mismatches (except C/C) to a defective hygromycin-resistant (Hyg^R^) gene reporter construct integrated into the chromosome. We find that the specificity of *M. smegmatis* mismatch repair *in vivo* matches that found previously for *T. kodakarensis in vitro*, suggesting a commonality in the NucS-promoted MMR systems in these bacteria. Finally, we used oligo-mediated recombineering to deliver defined mismatches to the defective hygromycin-resistant target in the chromosome and find that a cut induced by NucS at a G-G mismatch is processed by a 5′-3′exonuclease *in vivo*, or a 5′ flap endonuclease, which is limited in extent to no more than 8–10 base pairs, and that repair is independent of RecA, RadA, and non-homologous end joining functions Ku and LigD. A mechanism of mycobacterial NucS MMR is discussed.

## Materials and methods

### Bacterial strains and media

The *M. smegmatis* strains used in this study were derived from mc^2^155; the *M. tuberculosis* strain used in this study was H37Rv. *M. smegmatis* and *M. tuberculosis* were grown in 7H9 broth with 0.05% Tween 80, 0.2% glycerol and OADC (oleic acid-albumin-dextrose-catalase; Becton, Dickinson); transformants were selected on 7H10 plates with 0.5% glycerol and OADC with appropriate antibiotics. For measuring mismatch DNA repair specificity of NucS *in vivo*, *M. smegmatis* was transformed with pIR542, a Giles integration vector that contains a defective *hyg* resistance gene; insertion into the *M. smegmatis* chromosome was done by selection for zeocin resistance.

Deletion mutants of *M. smegmatis* were constructed by ORBIT (20) using *attB*-integrating plasmids pKM611 or pKM614, both of which contain a defective *hyg* gene (in opposite directions). *M. smegmatis* strains MGM199 (Δ*recA*) and MGM156 (Δ*Ku* and Δ*ligD*) were gifts kindly provided by Michael Glickman (21). For these strains, ORBIT was used to deliver pKM614 into the intergenic region between MSMEG_1397 and *rpsL*. In these and all other cases, the defective *hyg* gene was placed in the chromosome in a direction that allowed oligos used in this study to target the lagging strand template. Verification of each ORBIT-promoted deletion strain was done by PCR amplification of both junctions of the inserted plasmid and the chromosomal target site, as well as the absence of an amplicon using primers internal to the target gene (compared to WT cells); see Supplementary Figure S1. Constructions of *leuB* mutants containing single or two base pair indels into the *leuB* active site codon R101 were done using oligo-mediated recombineering as described previously (22), with the exception that the defective *hyg*gene was supplied by pKM585 instead of pKM427. When needed, the following antibiotic concentrations were added to LB plates, 7H9 media or 7H10 plates: kanamycin (20 μg/ml), streptomycin (20 μg/ml), hygromycin (50 μg/ml), and zeocin (25 μg/ml). A list of mutants constructed and used in this study is shown in Table 1. PCR verifications of deletion mutants generated and used in this study are listed in Supplementary Figure S1.

**Table 1.** Strains used and constructed for this study

Strain name	Genotype	Description	Markers	Source
MC^2^-155	wild type	Efficient plasmid transformation (ept)	none	lab strain
MGM199	Δ*recA*	Deletion of *recA*	none	(21)
MGM153	Δ*Ku*, Δ*ligD*	Deletion of *Ku* and *ligD*	none	(21)
KM272	Wild type; defective *hyg* gene	MG1655/pKM461 (RecT); pKM614 (defective *hyg* gene) inserted into the MSMEG_1397-*rpsL* intergenic region by ORBIT	Kan^R^ Zeo^R^	This study
KM274	Δ*recA;* defective *hyg* gene	MGM199/pKM461 (RecT); pKM614 (defective *hyg* gene) inserted into the MSMEG_1397-*rpsL* intergenic region by ORBIT	Kan^R^ Zeo^R^	This study
KM295	Δ*Ku*, Δ*ligD; d*efective *hyg* gene	MGM153/KM461 (RecT); pKM614 (defective *hyg* gene) inserted into the MSMEG_1397-rpsL intergenic region by ORBIT	Kan^R^ Zeo^R^	This study
KM165	Δ*nucS*::pKM464	MC^2^-155 deleted of MSMEG_4923 by ORBIT	Hyg^R^	This study
KM183	Δ*nucS*::*attP*	KM165 - cured of pKM464	none	This study
VF20	Wild type; defective *hyg* gene	MC^2^-155/pKM461 (RecT); pIR542 (defective*hyg* gene) inserted into phage Giles site	Kan^R^ Zeo^R^	This study
VF22	Δ*nucS*::*attP; d*efective *hyg* gene	KM183/pKM461 (RecT); pIR542 (defective *hyg* gene) inserted into phage Giles site	Kan^R^ Zeo^R^	This study
KM209	Δ*fenA*::pKM464	MC^2^-155 deleted of MSMEG_3883 by ORBIT	Hyg^R^	This study
KM210	Δ*fenA*::*attP*	KM209 - cured of pKM464	none	This study
KM289	Δ*fenA*::pKM611 (defective *hyg* gene)	KM210/pKM461 (RecT); pKM611 (defective *hyg* gene) inserted Δ*fenA::attP* site	Kan^R^ Zeo^R^	This study
KM215	Δ*radA*::pKM464	MC^2^-155 deleted of MSMEG_6079 by ORBIT	Kan^R^ Zeo^R^	This study
KM284	Δ*radA*::*attP*	KM215 - cured of pKM464	none	
KM287	Δ*radA*:pKM611 (defective *hyg* gene)	KM284/pKM461 (RecT); pKM611 (defective *hyg* gene) inserted Δ*radA::attP* site	Hyg^R^	This study
KM288	Δ*recA*, Δ*radA*:pKM611 (defective *hyg* gene)	MGM199/pKM461 (RecT); pKM611 (defective *hyg* gene) inserted Δ*radA* gene by ORBIT	Kan^R^ Zeo^R^	This study
KM229	MSMEG_2379 (*leuB* frameshift)	Two bp deletion in the*leuB* arginine-101 codon; pKM461; pKM585	Kan^R^ Strep^R^	This study
KM230	MSMEG_2379 (*leuB* frameshift)	One bp deletion in the *leuB*arginine-101 codon; pKM461; pKM585	Kan^R^ Strep^R^	This study
KM231	MSMEG_2379 (*leuB* frameshift)	One bp insertion in the*leuB*arginine-101 codon; pKM461; pKM585	Kan^R^ Strep^R^	This study
KM239	MSMEG_2379 (*leuB*frameshift)	Two bp deletion in the leuB arginine-101 codon; pKM461; cured of pKM585	Kan^R^	This study
KM321	NucS-Y12A	Binding site mutant of *M. smegmatis* NucS	none	This study
KM322	NucS-N48G	Shows wild type levels of MMR	none	This study
KM323	NucS-W49A	Binding site mutant of *M. smegmatis* NucS	none	This study
KM262	NucS-D138A	Active site mutant of *M. smegmatis* NucS	none	This study
KM263	NucS-E152A	Active site mutant of *M. smegmatis* NucS	none	This study
KM264	NucS-K154A	Active site mutant of *M. smegmatis* NucS	none	This study
Mtb-25	MtbΔ*nucS*::pKM488	H37Rv deleted of Rv1321 by ORBIT; pKM461	Kan^R^Hyg^R^	This study

### Plasmids

Oligo-recombineering plasmid pKM402, Che9 RecT and Bxb1 Integrase-expressing plasmid pKM461, and ORBIT integration plasmids have been described previously (20,23) and are currently available at the Addgene plasmid depository. The phage Giles integration vector pGH1000A contains the phage *attP* site and integrase allowing for easy integration of the plasmid into the *M. smegmatis* chromosome (24) and was kindly supplied by Graham Hatfull. This plasmid integrates into an *attB* site near the 3′ end of the tRNA^Pro^ gene (MSMEG_3734). Plasmid pKM497 is a derivative of pGH1000A that contains the PgroEL promoter, a multiple cloning site, and carries a streptomycin resistant cassette in place of the *hyg* gene (plasmid map shown in Supplementary Figure S2A). Plasmid pIR540 is a derivative of pKM497 that expresses full-length *M. smegmatis nucS* gene (MSMEG_4923) from the PgroEL promoter (see Supplementary Figure S2B). Plasmid pIR541 is similar to pIR540 except that it is missing the last five codons of the *nucS* gene. Plasmid pIR542 is a Giles integration vector (Zeo^R^) that contains a defective hygromycin-resistant gene where the codon for glycine 110 (GGA) has been altered to stop codon (TAG). Plasmid pKM611 is a Bxb1 *attB*-containing ORBIT-integration vector that contains the same defective hygromycin-resistant gene from pIR542; pKM614 is the same as pKM611, but with the defective-hygromycin-resistant gene in the opposite direction relative to the Bxb1 *attB* site. Plasmid pKM585 (see Supplementary Figure S2C) contains a Bxb1 *attP* site, the defective *hyg* gene, and integrates into the endogenous Bxb1 *attB* site of *M. smegmatis* with the help of pKM461 (RecT, Bxb1 Integrase). Plasmids and sequences of pKM487, pIR540 and pKM585 will be made available by depositing them in Addgene plasmid depository. Details of other plasmid constructions, maps, and sequences are available upon request.

### Oligonucleotides

Oligonucleotides used for the determination of mismatch specificity are listed in Supplementary Table S1. Oligonucleotides (188 mers) used for mutant construction by ORBIT are listed in Supplementary Table S2 and were obtained from IDT as Ultramers at a concentration of 100 μM (delivered in 96-well plates or tubes). They were supplied desalted with no further purification and diluted 10-fold in sterile ddH_2_O. Final concentrations (250 to 350 ng/ml) were determined by absorbance maxima at 260 nm (Abs_260_). Oligos (70–74 mers) used in RecT-promoted recombineering experiments for the repair of the defective *hyg* gene were obtained from Life Science Technologies and are listed in Tables S1 and S2. The position of the base restoring hygromycin resistance in these oligos was typically located at the central position (base 35); 1–2 μg of each oligo was added to electrocompetent cells for SNP transfer.

When oligos were used to create two mismatches consisting of a non-repairable C-T mismatch (conferring Hyg^R^) and a repairable mismatch (G–T or G–G), the two bases creating the mismatches were at least 24 bases from the 5′ and 3′ ends of the oligo, respectively. This was important to prevent the mismatching bases from being processed by endogenous nucleases after annealing to the lagging strand template, which is mostly restricted to bases close to the ends of the oligo (25). To demonstrate that bases positioned 24 bases from the ends of oligos were not processed by endogenous nucleases either before or after being annealed to the lagging strand template, we show that oligos containing the Hyg^R^-restoring base positioned 24 bases from either the 5′ or 3′ ends of oligos, respectively, generated the same frequency of Hyg^R^ recombinants as one where the Hyg^R^-restoring base was at the central position; see Supplementary Figure S3.

### Transformations

Details of the transformation procedure using the ORBIT technology for mutant strain construction have been described previously (20). Electroporations for oligonucleotide-promoted recombineering experiments, here to restore the defective *hyg* gene, have been described previously (22). Briefly, 150 μl of a fresh overnight culture of *M. smegmatis* containing pKM461 (and integrated plasmids pIR542, pKM611 or pKM614 containing a defective *hyg* gene) was added to 20 ml of 7H9-OADC-tween containing 20 μg/ml kanamycin and swirled at 37°C overnight. The following day, when the culture reached an Abs_600_ of 0.5, anhydrotetracycline (Atc) was added to a final concentration of 500 ng/ml. In cases where the culture went past an absorbance of 0.5, it was diluted back to 0.5 and Atc was added. The culture was allowed to grow for an additional three hours or until the O.D. reached 1.0. Cells were swirled on ice for 10 min, collected by centrifugation, and washed twice with 20 ml of cold 10% glycerol. After the final centrifugation, the cells were resuspended in 2 ml 10% glycerol and kept on ice. Oligonucleotides (2 μg, unless otherwise specified in legends) were placed in Eppendorf tubes and 380 μL of electrocompetent cells were mixed with oligos, then transferred to 0.2 cm electroporation cuvettes. Cells were shocked and transferred to 2 ml 7H9-OADC-tween and grown overnight at 37°C as described previously (20). Appropriate dilutions of overnight cultures were plated on LB or 7H10-OADC plates (±50 μg/ml hygromycin) to determine the fraction of Hyg^R^ transformants.

### Mutagenicity assay

The lack of NucS repair activity in *M. smegmatis* strains was determined by picking a single colony from a 7H10-OADC plate, inoculating it into a 5 ml culture of 7H9-OADC-tween, and growing overnight to a final O.D. between 1.8–2.5. Aliquots (150–200 μl) were then spread on 7H10 plates containing 150 μg/ml of rifampicin; total cell numbers were determined by plating dilutions of the culture on 7H10-OADC plates. Plates were incubated for 4–5 days at 37°C. The frequency of spontaneous mutation was determined by the titer of rifampicin-resistant colonies divided by the total cell titer.

### Recombineering with oligonucleotides generating two mismatches

To examine the extent of resection after NucS cutting, we designed thirteen oligos to create G–G and C–T mismatches separated by 2 to 32 base pairs along the chromosome, allowing us to measure the extent of processing of the C–T mismatch as a function of the distance (and position) from the NucS-proposed dsDNA cut (or nick) at the G–G mismatch. The sequences of these oligos and the position of the G-G mismatch relative to the C–T mismatch in each oligo (following annealing to the lagging strand template) are described in Figure Figure 7A. These oligos were electroporated into *M. smegmatis* containing pKM461 following induction with anhydrotetracycline, as described above. After overnight growth, portions of the culture were plated on 7H10 and 7H10 plates with 50 μg/ml hygromycin to determine the frequency of Hyg^R^ transformants. For the experiment done in mutant backgrounds, eight colonies from each transformation were picked and used to amplify the *hyg* region by PCR to determine the fate of the G–G mismatch by DNA sequencing.

## Results

### Mutator phenotype of *M. smegmatis nucS* strain and testing of the β-clamp interaction domain

**Figure 1.**
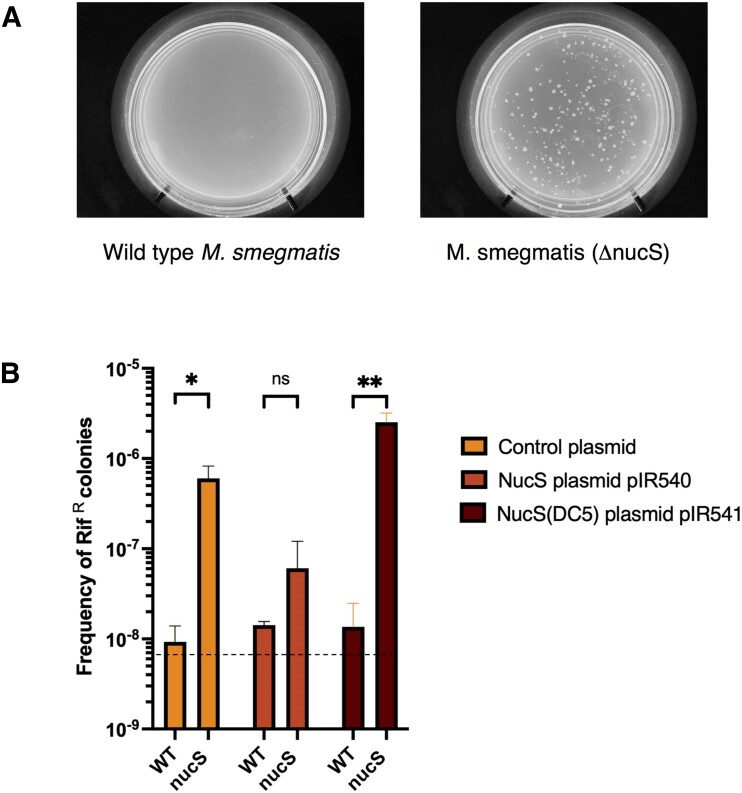
Mutagenic phenotype of *M. smegmatis* Δ*nucS* strain. (**A**) Wild type and a Δ*nucS* derivative of *M. smegmatis* were grown in 7H9-OADC-tween to saturation (O.D. of 2.0) and 200 μl of cultures were spread on 7H10-OADC-tween plates containing 150 μg/ml rifampicin. (**B**) *M. smegmatis* wild type and Δ*nucS* strains containing a control plasmid (pKM497), pIR540 (expressing full-length NucS), or pIR541 (expressing NucS containing a 5 amino acid truncation from the C-terminus, NucS(DC5)) were grown to saturation and plated on 7H10-OADC-tween rifampicin plates as described above. Dotted line represents the limit of detection. The mutation frequency is the titer of rifampicin-resistant mutants divided by the total number of cells plated. Data represent the means ± SD from three biological replicates; two-tailed *t*-test was performed (*P< 0.05; **P< 0.005).

Using the previously described ORBIT method for the generation of chromosomal modifications in mycobacteria (see Materials and methods), a deletion mutant of *M. smegmatis nucS* gene (MSMEG_4923) was constructed. The frequency of spontaneous mutation to rifampicin resistance was measured in both wild type *M. smegmatis* and its Δ*nucS* derivative (see Figure 1). As reported previously (19), an *M. smegmatis* strain deleted of *nucS* exhibited a mutator phenotype, with a >50–100-fold increase in the frequency of spontaneous mutations when compared to a wild type strain.

In complementation experiments, the expression of NucS from pIR540 reduced the mutation frequency considerably (about ∼15-fold), close to that observed in WT cells. However, a similar construct expressing a NucS protein with the last five amino acids deleted from the C-terminus (NucS-DC5) failed to complement the *nucS* strain, resulting in high levels of mutagenesis (see Figure 1B). This 5 amino acid C-terminal sequence of *M. smegmatis* NucS (EYRLF) is homologous to the C-terminal region in *P. abyssi* NucS that has been shown to interact with the PCNA sliding clamp, thus called the PIP domain (for PCNA interacting protein) (12). This region is also similar to the C-terminal 5 amino acids of NucS from the bacterium *C. glutamicum* (ELTLF), which required an interaction with the β-sliding clamp to promote dsDNA cutting of mismatched-containing substrates *in vitro* (17,18). This peptide in eubacteria has been generally referred to as the β-clamp binding domain, here shortened to β−CBD. The failure of the *M. smegmatis* NucS-DC5 to suppress the mutagenic phenotype of the Δ*nucS* mutant suggests that an interaction of the β−CBD in *M. smegmatis* NucS with the β-sliding clamp at the replication fork is required for efficient NucS-promoted cutting *in vivo*, and subsequent MMR.

It is possible that the loss of the C-terminal 5 amino acids causes *M. smegmatis* NucS protein to become unstable resulting in a mutagenic phenotype. To address this issue, we performed ORBIT to tag *nucS* to be able to follow the protein and its truncated version by Western analysis. However, C-terminal chromosomal fusions of wild type *nucS* with either Flag-DAS or GFP tags resulted in strains with mutagenic phenotypes (i.e. similar numbers of colonies on rifampicin plates to those seen with the Δ*nucS* strain), presumably by interfering with the interaction of the NucS β−CBD. Tagging of chromosomal *nucS* at the N-terminus was then performed using a 6x-His tag, but this modification also resulted in a strain that exhibited a mutagenic phenotype, suggesting the His tag may have interfered with dimerization of NucS or the binding of a protein partner *in vivo*. These results indicate that an interaction between a *free-ended* C-terminus β-CBD of *M. smegmatis* NucS and the β-clamp is required for efficient mismatch repair *in vivo*, and likely explains the inability of purified *M. smegmatis* NucS alone to recognize and cut mismatched dsDNA substrates *in vitro*, as was observed in a previous study (19). Biochemical analysis of NucS with mycobacterial DnaN β-clamp and mismatched substrates will be needed to verify this supposition.

### Mismatch DNA repair specificity *in vivo*

Previous mutation accumulation studies in both *C. glutamicum* and *M. smegmatis* have shown that the absence of the NucS MMR system results in an a greatly elevated rate of transitions, both G:C > A:T and A:T > G:C (the latter one being the higher of the two, opposite to what is observed in WT cells). In particular, Castañeda-García *et al* (26) used an integrated plasmid reporter system to measure the mutation rates (and specificities) of both transitions and transversions using mutant versions of *aph* (*kan*^R^) alleles. With their reporter strains, the fold increase in mutation rates (Δ*nucS*/WT) was highest (65-fold) for transitions that required repair of a G–T mismatch to generate a Kan^R^ cell. In contrast, the fold increase in transversion mutations on their reporter plasmids was low, including ones that are predicted to generate G–G and T–T mismatches (2.4- and 3.6-fold respectively). However, these mismatches are recognized and cut efficiently by *C. glutamicum* NucS *in vitro*. The low rate of NucS-dependent activity on transversion repair, despite efficient cutting of G–G and T–T mismatches *in vitro*, may be simply due to the low levels of G–G and T–T mismatches that arise during DNA replication in mycobacteria. Or it may mean that NucS does not act on these mismatches at high efficiency *in vivo*, despite highly efficient cutting of them *in vitro*.

**Figure 2.**
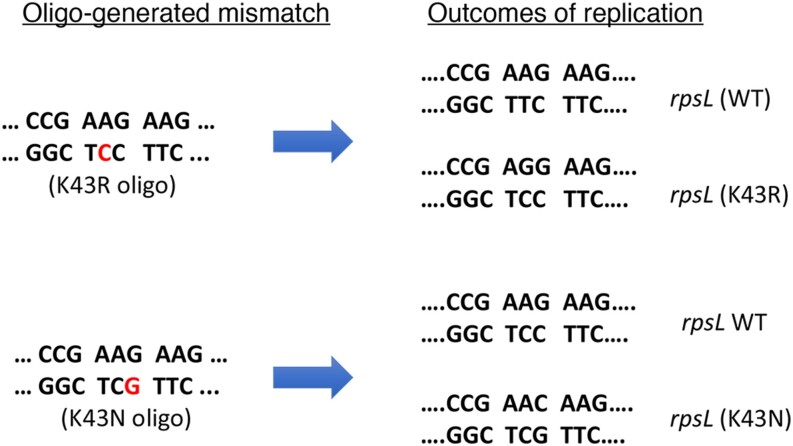
Oligo-mediated recombineering of the *rpsL* locus of *M. smegmatis*. (Left) Sequences of the oligo-generated mismatch in the *rpsL* gene resulting in a mismatch (A-C) predicted not to be recognized by mycobacterial NucS, and a mismatch (G–G) expected to be acted upon by NucS, as predicted from *in vitro* results of *C. glutamicum* (17,18). Mismatched bases delivered by the oligos are shown in red. (Right) Following replication of mismatched-containing DNA, both wild type and mutant versions of *rpsL* are generated. Both mutants are known to cause streptomycin resistance in mycobacteria (28).

We were thus encouraged to set up a similar assay to examine the mycobacterial mismatch repair system *in vivo* to compare the specificity of mismatch recognition to what has been reported *in vitro*. We thus tested the ability of Che9 RecT-mediated oligonucleotide recombineering to generate defined mismatches in a way that would allow us to follow the fate of mismatches by simple plating assays. A first test of this system was to deliver mismatches to the replication fork within the *rpsL* gene (22,27). Oligos were designed to generate either K43R or K43N changes to the *rpsL* ribosomal protein, both of which are known to confer resistance to streptomycin (28). The K43R oligo creates a C-A mismatch at the replication fork, while the K43N oligo generate at G-G mismatch (see Figure 2). According to mismatch specificity reported for *C. glutamicum* NucS *in vitro*, the K43R oligo would create a mismatch in *M. smegmatis* that would not be subject to NucS-mediated repair (C–A) and thus generate high levels of streptomycin-resistant colonies. Along the same lines, the K43N oligo would generate a mismatch that is subject to repair (G-G) and thus fail to generate streptomycin-resistant colonies. These predictions were fulfilled by the data shown in Table 2, showing that recombineering can be used to deliver mismatches to test *in vivo* specificities, and that *M. smegmatis* shows a similar mismatch repair specificity as exhibited *in vitro* by actinobacteria, but do so *in vivo*. The same experiment was performed in *M. tuberculosis* with similar results (Supplementary Figure S4).

**Table 2.** Frequencies^a^ of oligonucleotide-mediated streptomycin resistance in *M. smegmatis*

Strain	K43R oligo (A/C mismatch)	K43N oligo (G/G mismatch)
Wild type	1.59	0.0097
Δ*nucS*	1.05	1.4

^a^Titer of streptomycin resistant colonies/total colonies (× 10^−4^)

To further test the *in vivo* specificities of mismatch repair, a second assay was designed to study all the other types of mismatches. For this purpose, we inserted a stop codon (TAG) in place of the glycine codon (GGA) at position 110 in the hygromycinB phosphotransferase gene (*hyg*) in plasmid pIR542, a Giles integrating plasmid. The G110 residue was selected because of its presence in an extended loop on the surface of the protein (see Supplementary Figure S5A). In addition, the G-110 residue is not conserved among *hyg* genes from other bacterial species (Supplementary Figure S5B), highlighting this position as one that is likely tolerant to multiple types of amino acid substitutions. This prediction was fulfilled in the course of these assays.

Oligos were designed to convert the stop codon in the integrated plasmid pIR542 to various sense codons, allowing for full translation of the *hyg* gene. Each oligo generates a different type of mismatch (see Supplementary Table S1). If a mismatch escapes repair, it leads to a recombinant that expresses a functional phosphotransferase and generates Hyg^R^ colonies; on the contrary, if the mismatch is repaired, no or few Hyg^R^ transformants would be observed.

**Figure 3.**
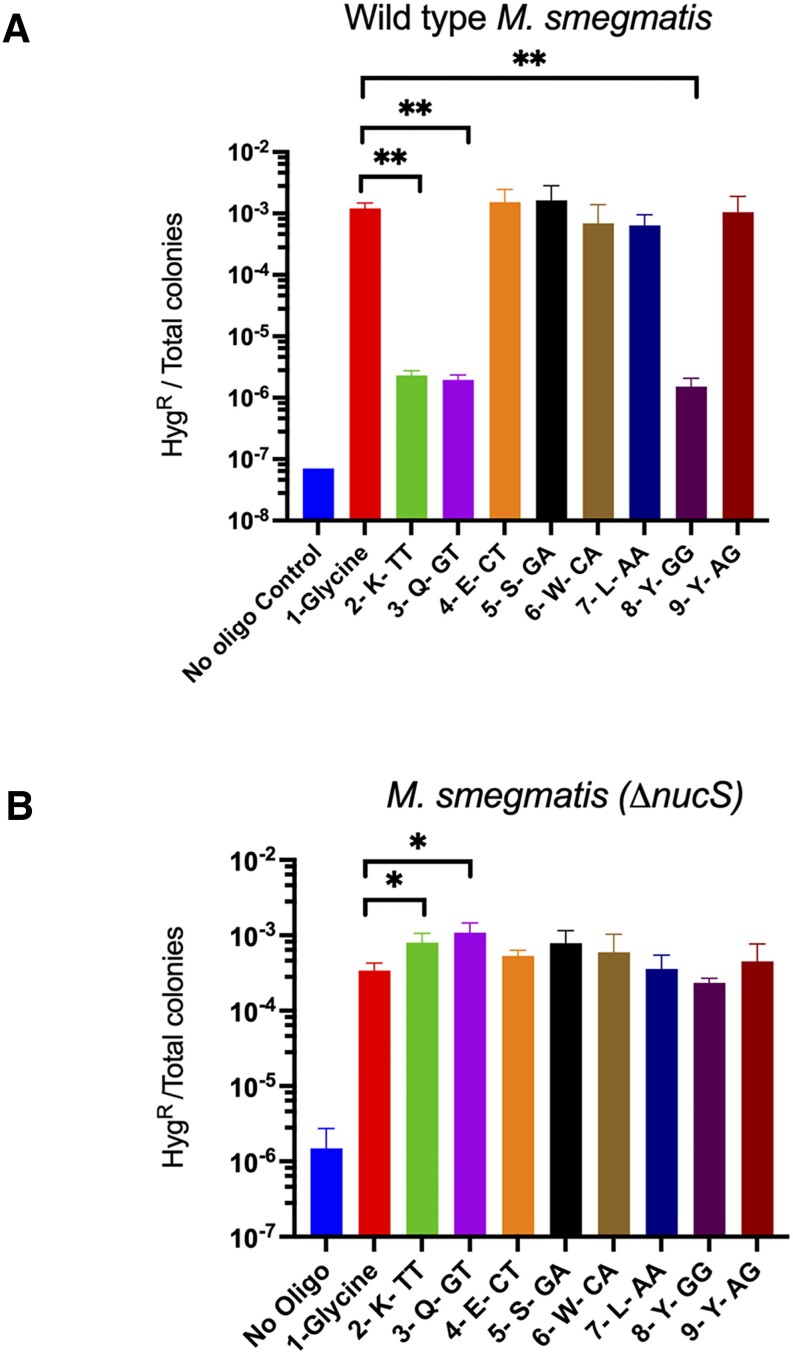
Mismatch recognition specificity of *M. smegmatis* NucS *in vivo*. Oligo-mediated recombineering experiments were carried out in WT (**A**) or Δ*nucS*(**B**) *M. smegmatis* cells containing RecT-producer pKM402 and a defective hygromycin gene, where codon-110 for glycine (GGA) had been altered to a stop codon (TGA). The oligos used generated 7 different mismatches for restoration of the *hyg* gene. Repair and non-repair of the mismatches are evident by the appearance of low and high frequencies of hygromycin-resistant colonies, respectively. Experiments were done in triplicate. Data represent the means ± SD from three biological replicates; two-tailed *t*-test was performed comparing results from the oligo that restores the original glycine codon to position 110 (red bar) to all other oligos that created the defined mismatches; *P< 0.05; **P< 0.005. Only significant *P* values are shown.

*M. smegmatis* containing pKM402 (RecT producer) and pIR542 were electroporated with the oligos listed in Supplementary Table S1 and the outgrowths were plated on LB plates with and without hygromycin, as described in Materials and methods; the results are shown in Figure 3. In otherwise wild type *M. smegmatis*, oligos that generated G-G, G-T and T-T mismatches generated Hyg^R^ colonies at more than a 500-fold lower frequency relative to transformations with oligos that generated all the other types of mismatches, indicative of repair of G–G, G–T and T–T mismatches *in vivo*. All the other mismatches tested escaped MMR, generating Hyg^R^ colonies at frequencies 10,000-fold greater than the control transformation with no oligo (Figure 3A). When the same experimental protocol was performed in the *M. smegmatis* Δ*nucS* strain, transformation with all the oligos generated high frequencies of Hyg^R^ colonies, revealing that in the absence of MMR, all mismatches generated were incorporated into the chromosome (Figure 3B). Out of the three mismatches recognized by NucS, the G–G mismatch, while not repaired, exhibited a bit lower frequency of Hyg^R^ colonies relative to the T-T and G-T mismatches in the Δ*nucS* background (Figure 3B). This could be due to an alternative repair mechanism that acts weakly on G–G in the absence of NucS (e.g. excision repair, or a weak unknown mechanism). These results reveal that *M. smegmatis* shows the same mismatch repair specificity *in vivo* as the archaeal TKO and the actinobacterial *C. glutamicum* strains exhibited *in vitro*, indicating that these MMR systems recognize identical types of mismatches and likely behave by similar mechanisms. Additionally, the G–T mismatch recognition specificity revealed here is consistent with the mutational specificity of *M. smegmatis* reported by Castaneda-Garcia *et al.* (26), who showed a high rate of transitions when the reporter plasmids generated G-T mismatches in order to restore kanamycin resistance. On the other hand, G–G and T–T (which if unrepaired lead to transversions), do not show high levels of NucS-dependent repair in mutation accumulation studies, even though NucS recognized and repaired G–G and T–T mismatches *in vivo* at the same rate as G-T mismatches (Figure 3A). This result is further elaborated on in the Discussion section.

### Inability of *M. smegmatis* NucS to repair small indels

**Figure 4.**
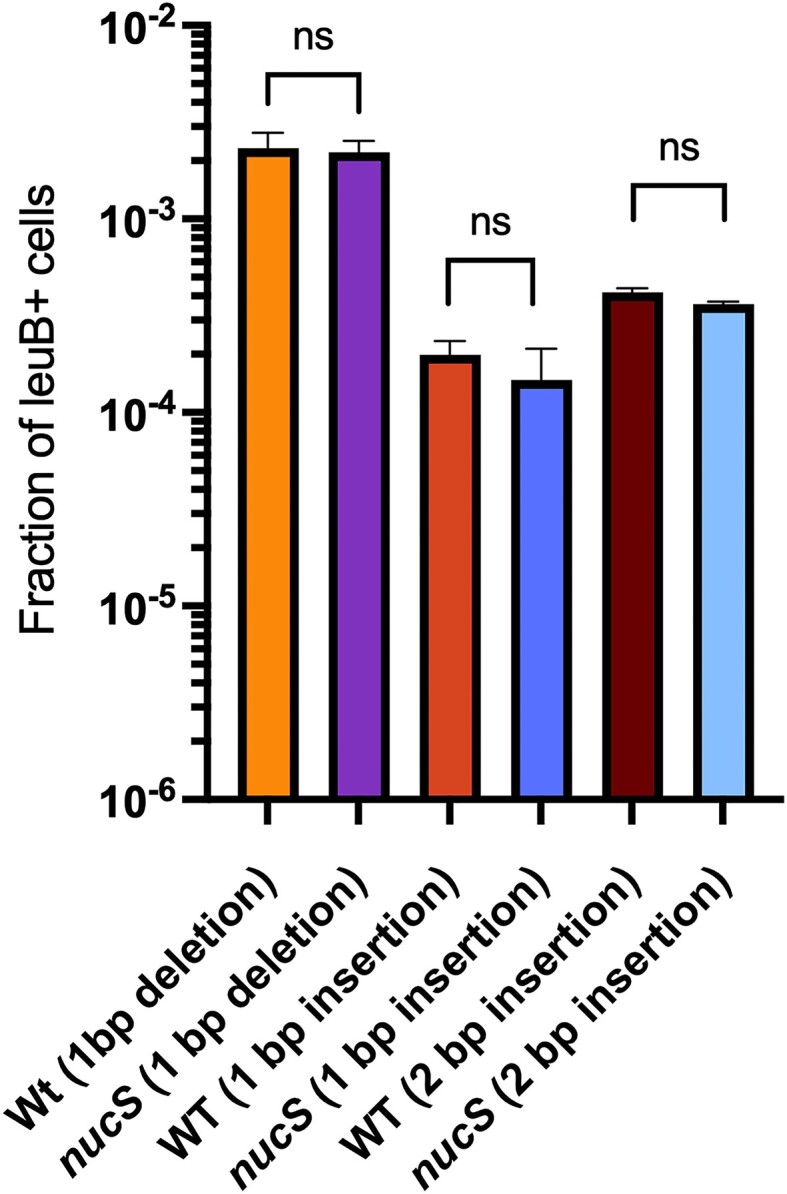
NucS does not repair indels. Oligo-mediated recombineering was performed in both *M. smegmatis* wild type and Δ*nucS* strains where the *leuB* gene had been deactivated by the deletion of 1 or 2 bp, or by the insertion of 1 bp in the codon that encodes for the active site residue Arg-101. An oligo containing the wild type *leuB* sequence was electroporated into these *leuB* cells which expresses the RecT annealase from plasmid pKM461. Overnight growth following electroporation was performed in 7H9 medium with leucine supplementation (50 μg/ml). LeuB + recombinants were measured by plating the cells on 7H10-OADC-tween plates without leucine supplementation; total cells were titered on 7H10-OADC-tween plates with 50 μg/ml leucine. Data represent the means ± SD from three biological replicates: Student's two-tailed *t*-test, *P< 0.05, **P < 0.005.

A test to examine the *in vivo* repair capacity of *M. smegmatis* NucS on small indels was done with our recombineering assay, examining the ability to remove 1–2 bp insertions and deletions in the *leuB* gene. Such indels inactivate *leuB* activity and recombineering with oligos that removed these indels allow these strains to grow on 7H10 plates without leucine supplementation. If NucS is inactive on DNA containing 1–2 bp indels, such oligo-mediated leuB^+^ strains will be easily observed. This was, in fact, the case, as insertions and deletions between 1–2 bp were easily removed from these *leuB* frameshift mutants using an oligo that contained the wild type *leuB* sequence, restoring the defective *leuB* allele back to wild type at high frequencies; this activity was independent of *nucS* (see Figure 4). The lack of activity of mycobacterial NucS on indels in this recombineering assay is in agreement with the MA experiments and reporter plasmids previously described (26). Also, the inability of NucS to repair 1–2 base indels (unlike *E. coli’*s MutS MMR system, which can repair 1–4 bp insertion loops) is consistent with the use of small insertions/deletions having a role in mycobacterial phase variation, where the inability to efficiently remove indels from chromosomal homopolymeric tracts contributes to silencing and controlling genes over an evolutionary timescale (29). This result, and those in Figure 3, further reveal that delivery of DNA mismatch to the chromosome by oligo-mediated recombineering faithfully mimics *bona fide* DNA mismatches that occur in the replication fork *in vivo*.

### Identification of NucS nucleolytic active site and mismatch recognition residues

**Figure 5.**
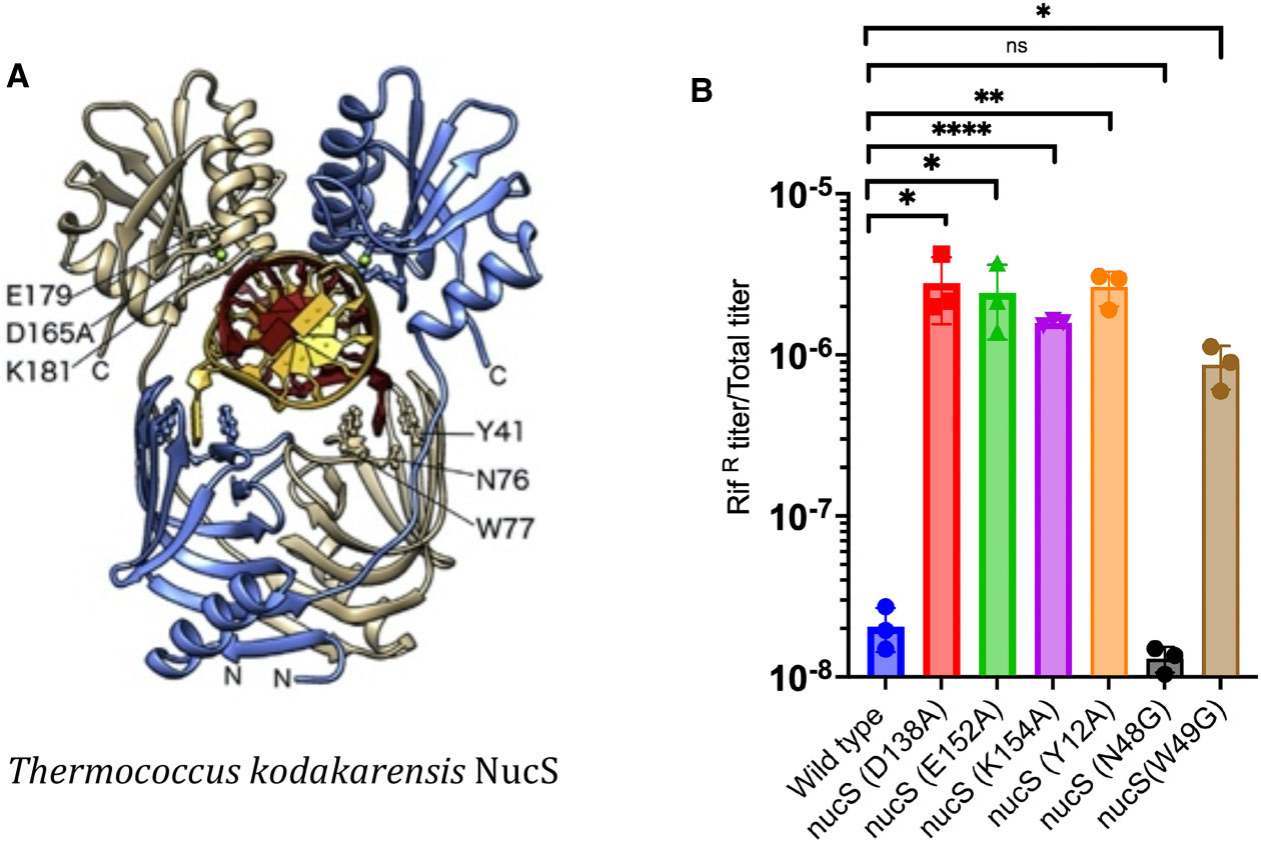
Mutations suspected of being critical for exonucleolytic activity and mismatched DNA binding in NucS are examined for MMR activity in vivo. (**A**) The structure of the NucS protein from *Thermococcus kodakarensis* is shown, highlighting the residues thought to be critical for exonucleolytic activity (E179, D165 and K181) and mismatched DNA recognition (Y41, N76 and W77). (**B**) The corresponding residues in *M. smegmatis* NucS (See Supplementary Figure S6 and text for details) were changed to alanine or glycine by recombineering and tested for levels of spontaneous mutation to rifampicin. Data represent the means ± SD from three biological replicates: Student's two-tailed *t*-test, *P< 0.05; ****P< 0.0001. The *T. kodakarensis* NucS structure was reprinted from Nakae *et al.* (14) with permission.

Nakae *et al.* (14) solved the structure of the NucS protein from *T. kodakarensis* and identified three amino acid residues that may be involved in nucleolytic activity (Figure 5A, left). In comparison with the *T. kodakarensis* NucS protein sequence, the corresponding amino acids in *M. smegmatis* NucS are D138, E152 and K154 (see Supplementary Figure S6). Using Che9 RecT-mediated oligo recombineering, each of these residues was separately changed to alanine and the subsequent strains were tested for mutagenic activity by plating on 7H10 plates containing rifampicin. The D138A, E152A and K154A mutations each conferred a mutagenic phenotype to *M. smegmatis*, implicating all three residues in NucS endonucleolytic activity (Figure 5B). Further confirmation of these residues as nucleolytic active site residues will require biochemical analysis of the mutant proteins.

In addition, Nakae *et al.* (14) proposed three residues that are involved in NucS binding to the mismatched bases based on the TKO NucS crystal structure complexed with dsDNA containing a G-T mismatch. Residues Y41, N76, W77 are all present at the mismatched recognition site. In particular, Y41 and W77 participate in stacking interactions with mismatch bases, while N76 forms hydrogen bonds between both its main chain nitrogen (Asn76-N) and its side chain nitrogen (Asn76-Nδ2) and either G-O6 (or T-O4) mismatched bases. The corresponding residues in *M. smegmatis* NucS are Y12, N48 and W49 (see Supplementary Figure S6). Each of these residues were modified by oligo recombineering and the mutants were subsequently tested for their mutagenic phenotypes by measuring frequencies of spontaneous resistant to rifampicin (see Figure 5B). Strains containing the *M. smegmatis* NucS Y12A and W49A alterations clearly exhibited mutagenic phenotypes. However, the NucS N48G strain did not show an elevated mutation rate above that seen with the wild type strain. Apparently, the hydrogen bond seen between the Asn76-Nδ2 nitrogen and the mismatched base oxygen in TKO NucS-dsDNA structure is not critical for the stabilization of the active site; however, the main chain nitrogen at this amino acid position may still play a role. Importantly, these results go on to show that NucS can recognize mismatches *in vivo* without the need for an interacting partner.

### Resection of NucS-promoted cuts *in vivo* involves limited processing by a 5′-3′ exonuclease

**Figure 6.**
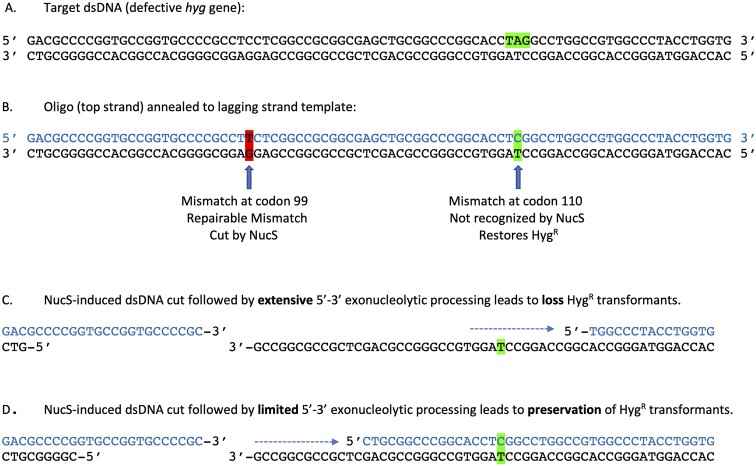
Description of a double mismatch-generating oligo. (**A**) The dsDNA sequence of the targeted region of the defective hygromycin-resistant gene (*hyg*). The GGA-110 codon has been altered to stop codon TAG (highlighted in green). (**B**). After annealing of the oligo (*Hyg repair-32* in blue type) to the target site in the *M. smegmatis* chromosome, the oligo creates two mismatches: a C–T mismatch (unresponsive to NucS) that leads to high levels of Hyg^R^ colonies and a T–G mismatch that is acted upon by NucS *in vivo*. (**C**) NucS is suggested to make a dsDNA cut at the mismatch. If the ends of the breaks are acted upon by a 5′-3′ exonuclease (action demonstrated by dotted blue arrows), Hyg^R^ recombinants will be lost if the processing extends to the C-T mismatch or beyond and removes the ‘C’. (**D**) If the processing by a 5′-3′ exonuclease is limited and does not extend to the C-T mismatch, high levels Hyg^R^ transformants will be generated.

The identification of nucleolytic active site residues in *M. smegmatis* NucS that lead to high mutability suggests that cutting *in vivo* likely occurs in the processing of mismatches in mycobacteria. Whether this involves a ssDNA nick (as seen with the MutSLH system) or a dsDNA break (as suggested by *in vitro* results from *C. glutamicum* NucS_Cg_) has not currently been established *in vivo*. In either case, we sought to examine the processing of such cuts by endogenous exonucleases *in vivo* by delivering two mismatches (generated by one oligo) to the chromosome via recombineering, with one mismatch (C–T) generating Hyg^R^ mutants (as described above) and the other mismatch (G–T) being recognized and cut by NucS. The target sequence is shown in Figure 6A. The oligo annealed to the template strand is shown in blue in Figure 6B and generates mismatches that are 32 bp apart on the lagging strand. It is assumed that exonucleolytic processing would occur from the NucS-induced cut at codon 99 to codon 110 of the Hyg^R^ gene. If processing by a 5′-3′exonuclease in the replicating strand (top blue strand in Figure 6C) proceeds past codon 110 of the Hyg^R^ gene, subsequent repair by resynthesis of the gap (or by recombinational repair) would lead to loss of the C-T mismatch and restoration of the stop codon. This, in turn, would lead to loss of Hyg^R^ transformants relative to the use of an oligo that did not generate a repairable mismatch (control oligo), but still generates the C-T mismatch that repairs the defective hyg gene. On the contrary, limited exonucleolytic processing from the G-T mismatch at codon 99 that did not proceed to codon 110 would not affect the level of Hyg^R^ transformants compared to the control oligo (Figure 6D).

This assay makes no assumptions regarding whether NucS makes a dsDNA cut or only a nick in the replicating strand, since processing of only the bottom strand (after a dsDNA cut) would not affect the levels of Hyg^R^ colonies (i.e. processing of the bottom strand in Figure 6B would remove the ‘T’ residue resulting in loss of the stop codon and conversion to Hyg resistance). In addition, while the assay shown in Figure 6 can examine 5′-3′ exonuclease functions, it is also capable of examining 3′-5′ exonuclease functioning of the top strand when the G–G mismatch is positioned downstream (3′) to the C–T mismatch (see below).

**Figure 7.**
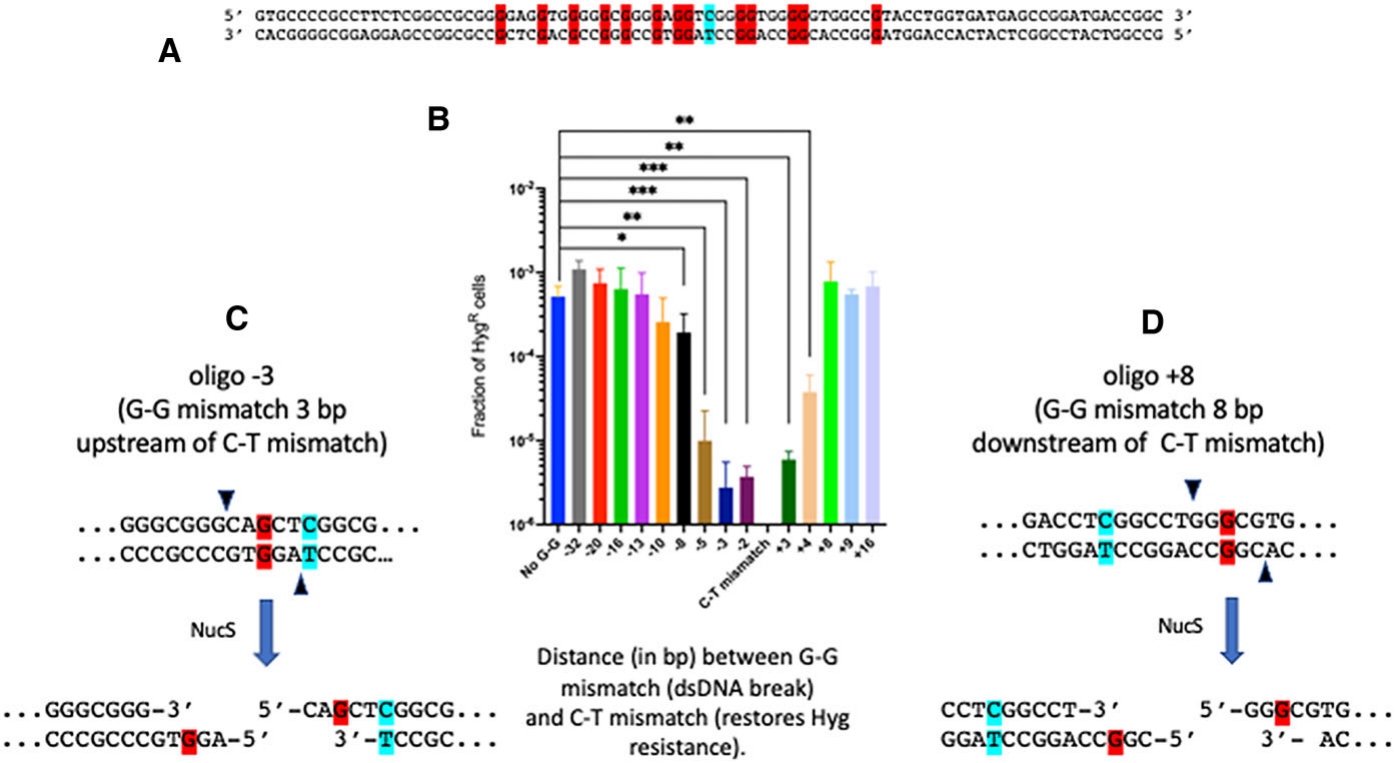
5′-3′ exonuclease processing of NucS-induced breaks. (**A**) Oligo (top strand) and template (bottom strand) are shown. The C–T mismatch (shown in cyan) leads to Hyg^R^ and is centered at position 44 of an 87 mer oligo (Hyg repair-CT). All other oligos used in this experiment are derivatives of this oligo and contain a G in place of C to create a G–G mismatches at 13 different positions (highlighted in red). Note that the ‘Gs’ generating the mismatches at the replication fork are at least 24 bases from the ends of the oligos to prevent processing by endogenous ssDNA nucleases. **(B)**. Oligo recombineering was caried out in *M. smegmatis* carrying the defective *hyg* gene and plasmid pKM461(RecT). Each of the 13 oligos used in this assay generates a C-T mismatch at a fixed position (i.e. the stop codon) and a G–G mismatch at variable positions as described in panel A; one oligo generates only the C-T mismatch (first column), while the –32 oligo creates a T–G mismatch 32 bases away from the C-T mismatch (second column). The names of the oligos on the X-axis define the number of base pairs between the two mismatches, and if the G-G mismatch is upstream (–) or downstream (+) of the C–T mismatch. The numbers of Hyg^R^ colonies and total cell number were determined by plating on 7H10-OADC-tween plates with and without hygromycin, respectively. Data represent the means ± SD from 3–5 biological replicates; two-tailed *t*-test was performed comparing results from the control oligo (no G-G mismatch, blue bar) to each test oligo; *P< 0.05, **P< 0.005, ***P< 0.001. Only significant P values are shown. (**C**) Diagram of a NucS-promoted dsDNA cut made by oligo -3 is shown. After cutting by NucS, the C of the C–T mismatch is 6 bases away from the 5′ end. (**D**) Diagram of a NucS-promoted dsDNA cut made by oligo +8 is shown. After cutting by NucS, the ‘C’ of the C–T mismatch is 6 bases away from the 3′ end.

A first test of this two-mismatch-promoting oligo (Hyg repair-32) showed that it could generate Hyg^R^ transformants at the same frequency as a control oligo that generated the C–T mismatch but not the G–T mismatch (∼10^−3^ Hyg^R^ transformants/total cell titer – see Figure 7B, second column). Sequencing of PCR fragments containing this region from 8 Hyg^R^ colonies found that all the G-T mismatches were repaired (resulting in a ‘C’ in the replicating strand). This result shows that exonucleolytic processing from the NucS-promoted cut at the G–T mismatch did not extend 32 bases to the C–T mismatch, leaving the frequency of Hyg^R^ colony formation intact relative to the control oligo (no G–G mismatch).

Given the result above, we performed the same test with a series of oligos that varied the distance between a repairable mismatch (this time G–G) and the C–T mismatch that confers Hyg^R^. Oligos generating both G–G and C–T mismatches were electroporated into *M. smegmatis* containing pKM461 (RecT) and the defective *hyg* gene; see Figure 7A for positions of the G–G mismatches relative to the C–T mismatch in each of these oligos. We followed the frequency of Hyg^R^ colonies from the outgrowth when the two mismatches were relatively close (2–5 bp) or further apart (16 or 20 bp), as well as oligos where the repairable mismatch was downstream of the C–T mismatch at variable distances (see Figure 7A). At a distance 20 bp upstream of codon 110, a G–G mismatch-guided NucS-promoted cut did not lead to loss of Hyg^R^ colonies relative to the oligo with no G-G mismatch. This result suggests that 5′-3′ processing of the predicted dsDNA cut in the top strand did not extend 20 bp to the C–T mismatch, which would have resulted in loss of Hyg^R^ colonies relative to the oligo with no G-G mismatch (like the result described above). On the other hand, when the G–G mismatch was only 5 bp away from the C–T mismatch, there was a 50-fold drop the frequency of Hyg^R^ colonies. Furthermore, when the G-G mismatch was only 3 bp away from the C–T mismatch, there was a ∼200-fold drop in Hyg^R^ colony formation. These results show that a dsDNA break (or nick) at the G–G site close to the C–T mismatch resulted in limited 5′-3′ exonucleolytic processing of the top strand that extended to the C–T mismatch, removing the ‘C’ base, with eventual restoration of an ‘A’ at this position to restore the stop codon (leading to loss of Hyg^R^ colonies) (see Figure 7B). Alternatively, the limited processing of cuts made at the G–G mismatch in vivo could also be due to a 5′-Flap endonuclease.

As mentioned above, when the position of the G–G mismatch was designed to be downstream of the C–T mismatches (five positions shown on Figure 7A), the assay can follow the exonucleolytic processing by a 3′-5′ exonuclease. In this case, we found that all these oligos, except for the ones positioned at the +3 and +4 positions, resulted in no loss in the frequency of Hyg^R^.

To compare the results of 5′-3′ versus 3′-5′processing in the assay shown in Figure 7, one must consider that the NucS proteins from *T. kodakarensis* and *C. glutamicum* have been shown to cut dsDNA and leave five base 5′ overhangs. This is likely true for mycobacterial NucS as well. To this point, the data in Figure 7 are expressed in a table format (see Table 3) as the number of bases that must be digested before processing leads to loss of Hyg^R^ transformants (i.e. before digestion to the ‘C’ residue in the C-T mismatch shown in Figure 7). This table assumes that the cutting *in vivo* results in the same 5′ five base overhangs that was observed when *C. glutamicans* NucS was examined *in vitro*, whether that cut involves a dsDNA break or a cut only in the newly synthesized strand (top strand in Figure 7A). The key observation is that loss of Hyg^R^ occurs at 285-fold higher frequency when 6 bases need to be digested by a 5′-3′ exonuclease (see -3 oligo in Table 3; see also Figure 7C) relative to when 6 bases need to be digested by a 3′-5′ exonuclease (see +8 oligo in Table 3; see also Figure 7D). This result reveals a much higher level of 5′-3′ exonucleolytic processing of these substrates *in vivo*. While processing on the 3′ strand is clear in Figure 7, it is only present when the number of bases to the ‘C’ residue is reduced to 2 bases (oligo + 4 in Table 3) or 1 base (oligo + 3 in Table 3). In both these cases, however, the Hyg^R^ frequency is 15-fold and 2-fold higher, respectively, (reflecting less processing to the C–T mismatch) when compared to the 6 bases that need processing on the 5′ strand (oligo –3). In addition, it is possible that the processing observed on the 3′ ends could be due to ‘limited nibbling’ of the 1–2 bases by non-specific nucleases.

**Table 3.** The loss of Hyg^R^ colonies following NucS cutting at the G-G mismatch (from Figure 7) as a function of the number of base pairs to excise the "C" residue from the C-T mismatch (that otherwise would lead to hygromycin-resistant colonies) from either the 5' or 3' direction. Relative Hyg^R^ frequencies shown are normalized to that seen with the -20 oligo

Oligo	# bases to ‘C’ from 5′ end	# bases to ‘C’ from 3′ end	Relative Hyg^R^ frequency
**−20**	23	−	1.0
**−16**	19	−	1.4
**−13**	16	−	1.2
**−10**	13	−	1.0
**−8**	11	−	0.37
**−5**	8	−	0.019
**−3**	6	−	0.005
**−2**	5	−	0.07
**+3**	−	1	0.011
**+4**	−	2	0.073
**+8**	−	6	1.5
**+9**	−	7	1.1
**+16**	−	14	1.3

These results support a model of 5′-3′ exonucleolytic processing of the dsDNA break at the site of a NucS-induced cut at a mismatch *in vivo*. What is also revealing is that while the data show a gradual increase in Hyg^R^ colonies as the G–G mismatch is moved from the –10 to the –13 position (though not statistically significant, the trend is clear), at 13 bases away, the frequency of Hyg^R^ transformants is equal to the frequency seen with the control oligo (i.e. no G-G mismatch). This result suggests that while there is 5′-3′ exonucleolytic processing from the NucS-induced cut, it is limited to a small patch of DNA and inconsistent with the ∼200 bp to 1 kb of processing of ssDNA that usually accompanies RecA-promoted dsDNA break repair events. Such short patch repair is reminiscent of a similar pathway of mismatch repair events promoted by MutS in *E. coli*. While these results have been presented in Figure 7 in the context of NucS making a dsDNA cut at the G–G mismatch, the same conclusions can be drawn if NucS promotes a nick in the replicative strand (blue strand in Figure 6B).

### Repair of NucS-promoted ds-DNA breaks is not dependent on RecA- or RadA-promoted homologous recombination, or NHEJ

The double-mismatch-promoting oligo assay described above can also be used to identify genetic functions that work downstream of NucS (e.g. genes like *recA*that would process a dsDNA break). For this assay, we used an oligo that promotes formation of the C–T mismatch that restores Hyg^R^ to the defective *hyg* gene and a G–G mismatch 20 base pairs away. In a wildtype cell, there is no effect on a C–T mismatch by a repairable G–G mismatch 20 bp away (see Figure 7). Furthermore, the G–G mismatch at the targeted codon is in the wobble position, so the amino acid residing at this position is independent of mismatch repair.

If a dsDNA cut is indeed generated *in vivo* at the G–G mismatch, and recombination repair acts on that break, the absence of RecA protein would be expected to lead to an unrepaired dsDNA break and death of that cell, resulting in loss of Hyg^R^ transformants relative to a WT host. Along the same line of reasoning, if the processing of the dsDNA cut (or nick) at the G-G mismatch was impaired by a mutation in a resident 5′-3′ dsDNA or flap endonuclease, it likely would lead to some level of interruption of the *hyg* reading frame by loss of base pairs other than 3 (or multiples of 3), again leading to loss of Hyg^R^ transformants relative to WT. And finally, if the missing function results in no exonucleolytic activity at all, and only a nick is formed by NucS *in vivo*, the nick could ultimately be repaired by ligation. Repeat cycles of cutting and religation might occur, but the ultimate result would lead to a viable cell that did not repair the mismatch but does exhibit a Hyg^R^ phenotype. Thus, in this experiment, we examined if the presence of the G–G mismatch influences the frequency of Hyg^R^ transformants, as loss of Hyg^R^ transformants in a mutant strain, compared to a control oligo not generating the G-G mismatch, would indicate involvement of that gene in mycobacterial MMR. In addition, 8 Hyg^R^ candidates from each mutant background were sequenced to determine if the G-G mismatch was indeed repaired or not.

**Figure 8.**
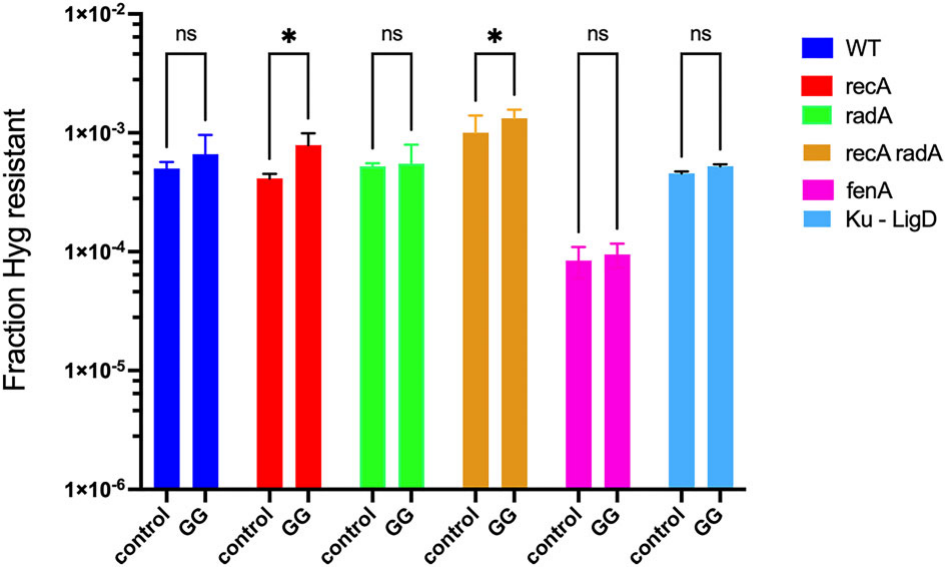
Testing of functions suspected of playing a role in *M. smegmatis* NucS-promoted MMR repair. Oligos generating C-T mismatches that promote Hyg^R^ and generate no other mismatch (control), or a G-G mismatch 20 bp away from the C-T mismatch (G–G), were electroporated into *M. smegmatis* strain mc2-155 and a series of deletion mutants in genes suspected of being involved in *nucS*-promoted MMR. All strains contained the RecT-producing plasmid pKM461. Disruptions of genes involved in NucS-promoted MMR are expected to generate low frequencies of Hyg^R^ colonies with oligos containing the G–G mismatch (G–G), compared to high frequencies of Hyg^R^ expected with oligos not containing the G–G mismatch (control oligo). Experiments were done in triplicate; standard deviations are shown. A 2-way ANOVA statistical analysis was performed comparing the frequencies of each mutant, with and without the G–G mismatch; *t*-test, *P< 0.05.

The most obvious gene to test for involvement of *M. smegmatis* MMR is *recA*, given the dsDNA breaks *in vitro* seen with NucS_Cg_. Besides RecA, the *M. smegmatis* RadA function was also tested in this assay. RadA is an archaeal analog of the RecA recombinase and can form nucleoprotein filaments on DNA and catalyze DNA pairing and strand exchange (30,31). RadA has a role in stimulating branch migration that extends heteroduplex formations in RecA-mediated strand transfer reactions (32,33). Mutants of RadA in *E. coli* have strong synergistic phenotypes with *recG* mutants, a branch migration enzyme, and show synthetic genetic interactions for survival to AZT when combined with several known helicases including PriA, RuvAB, UvrD, and others (34). *B. subtilis* RadA has also been shown to possess a 5′-3′ helicase activity capable of unwinding recombinational intermediates (35). The *M. smegmatis radA* gene seemed especially relevant to a study of *nucS* function since both these genes are of archaeal origin and in one genus of archaea (*Thermococcus*), *nucS* is co-transcribed with *radA* (12,13). Other functions in *M. smegmatis* were also tested in this assay and included a strain mutant in both Ku and LigD (21,36), to see if NHEJ had a role in mycobacterial MMR, and the MSMEG_3883 gene which encodes *fenA*, a flap endonuclease involved in processing of 5′ overhangs (37,38). Results of this experiment are shown in Figure 8. In these experiments, the frequency of Hyg^R^ transformants with the control oligo is compared to that seen with an oligo generating both the C–T (Hyg^R^) and a G–G mismatch 20 bases away, for each of the mutant strains tested.

In support of the data of limited resection shown in Figure 7, neither *recA*, *radA*, or the double mutant exhibited a loss in the frequency of Hyg^R^ transformants following transfer of the oligo generating the G–G mismatch relative to the control oligo (where a G-G mismatch is not generated). In *recA* hosts, there was a small stimulation of the frequency of Hyg^R^ cells. It is not known why this is the case, though it could be due to an inhibitory effect of RecA on NucS-promoted repair or may be the result of increased RecT-promoted annealing of the oligo in the absence of RecA. Nonetheless, since no loss of Hyg^R^ transformants is observed with these mutants, recombinational repair is not involved in NucS-promoted MMR. Further proof that MMR occurred in the absence of both RecA and RadA came from sequencing of the *hyg* gene from eight Hyg^R^ transformants from each of these genetic backgrounds, including the double mutant. Sequencing of the base which generated the G-G mismatch in both *recA* and *radA* host showed that 100% of the G-G mismatches (8/8) from each host had been efficiently repaired (See Table 4).

**Table 4.** Results^a^ of sequencing the G–G mismatched region from eight Hyg*^R^* recombinants from the experiment shown in Figure 8

Wild type	C	G
*recA*	8	0
*radA*	8	0
*recA radA*	8	0
*Ku ligD*	8	0
*fenA*	8	0
*nucS*	1	7

^a^Number of colonies (out of 8) containing a ‘C’ (repair) or a ‘G’ (non-repair) at the mismatch position generated by oligo “Hyg repair-20”.

As a control, when the assay was performed in a *nucS* host followed by colony PCR and DNA sequencing, the G/G mismatch did not get repaired in 7 colonies, as expected. However, we found 1 colony showing a ‘C’ in the position of the mismatch (Table 4). This residual repair is unlikely due to processing of the 5′ end of the oligo after annealing to the lagging strand template (see Supplementary Figure S3) but may be due or to the action of an alternate repair system (*e.g*. excision repair) that is active in the absence of NucS. Experiments are underway to examine this question. However, these results do reveal that NucS is involved in the majority (87%) of the MMR repair events and employing it in different genetic backgrounds can be used to identify functions that work downstream of *nucS*.

The results shown in Figure 8 also suggest that neither NHEJ functions LigD and Ku (MSMEG_5570 and MSMEG_5580) or the FenA flap endonuclease (MSMEG_3883) are involved in mycobacterial mismatch repair, as none of these functions led to loss of Hyg^R^ transformants with the oligo that generates the G-G mismatch relative to the control oligo. Again, sequencing 8 Hyg^R^ transformants from each of these hosts reveled that the G-G mismatch has been efficiently repaired *in vivo* (Table 4). The absolute frequencies of Hyg^R^ transformants is lower when performed in the *fenA* mutant strain relative to the other hosts, which is attributed to a possible effect on the frequency of oligo-mediated recombineering, given the role of FenA from *B. subtilis* which has recently been proposed to have a role in processing of Okazaki fragments (39). (This question was not further addressed in this study). Other functions thought to be important players in NucS-promoted MMR in mycobacteria, such as other dsDNA exonucleases and helicases, are currently being examined using this double-mismatch oligo-recombineering assay.

## Discussion

The discovery of the EndoMS/NucS function, principally found in archaea and Actinobacter species, revealed that a new pathway for DNA mismatch repair had been identified that does not follow the nearly ubiquitous paradigm of MutS DNA mismatch recognition, followed by MutL (or MutH) nicking of the duplex strand containing the ‘wrong’ base. Instead, *in vitro* characterizations of the NucS proteins from the archaeal species *T. kodakarensis* and the Actinobacter bacterium *C. glutamicum* revealed that while they recognized substrates containing mismatched bases, these proteins proceed to make cuts in both strands of the dsDNA substrates *in vitro*. The cutting of dsDNA containing mismatches with *T. kodakarensis* NucS showed that the cut left five nucleotide 5′ overhangs and recessed 3′ -OH ends leaving ligateable sticky ends characteristic of restriction enzymes. Further support of the dsDNA cutting capability of NucS comes from the Xray structural analysis of the NucS protein from *T. kodakarensis* which clearly has structural characteristics of a restriction enzyme: a homodimeric structure consisting of two domains, an N-terminal domain acting as a dimerization function that recognizes the mismatched bases that are flipped out (like restriction enzyme Ecl18kl), and a C-terminal domain that contains the active site residues for cutting dsDNA in a manner reminiscent of type II restriction enzymes (14). These studies strongly suggest that NucS acting in archaea and actinobacterial species act on mismatched bases by creating a dsDNA break at the site of the mismatch, though this cutting has not been demonstrated *in vivo*.

A first step to examine the capability of NucS in mycobacteria to promote such a dsDNA break was investigated by Castenda-Garcia *et al.* (19), who showed that an *M. smegmatis* Δ*nucS* strain showed a mutagenic phenotype when plated on rifampicin plates, which we confirmed here. However, their biochemical analysis revealed that while NucS bound to ssDNA, it did not bind to or cut substrates containing DNA mismatches, leaving open the possibility that NucS from mycobacteria might not behave in a similar manner to NucS from *C. glutamicum* (despite a 73% sequence identify between the two proteins). The most likely scenario for their inability to see cutting by *M. smegmatis* NucS was that, like NucS from *C. glutamicum*, it requires an interaction with its cognate β-clamp. To this end, our ability to complement the mutagenic phenotype of *M. smegmatis* Δ*nucS* with full-length NucS, but not one missing the last 5 amino acids containing the β−CBD, is consistent with this supposition. Verification of this interaction between *M. smegmatis* NucS and its β-clamp will require biochemical analysis of these functions. In addition, our mutational analyses of amino acid residues in *M. smegmatis* NucS corresponding to residues in TKO NucS predicted to be involved in mismatched-DNA binding and exonucleolytic cutting, reveal the importance of these residues in *M. smegmatis* NucS for mismatch repair.

We then leveraged the oligo-mediated recombineering capabilities of *M. smegmatis* to deliver defined mismatches directly to the mycobacterial chromosome to examine the mismatch specificity of NucS MMR. We found that mismatches containing G-T, G-G, and T-T were specifically repaired using the NucS MMR pathway, while none of the other mismatches were corrected. The mismatch recognition specificities revealed here *in vivo* are consistent with previous specificities shown in *C. glutamicum* NucS_Cg_*in vitro*. (17,18). They are also consistent with the mutational specificity of *M. smegmatis* reported by Castaneda-Garcia *et al* (26), who showed a high rate of both G:C > A:T and A:T > G:C transitions in *M. smegmatis nucS* strains (relative to wild type strains) using mutation accumulation experiments. This result agrees with the recognition of G–T mismatches by NucS *in vivo*, which would prevent the accumulation of transition mutations in wild type cells. However, while large increases in transversions are not observed in mycobacterial *nucS* mutant MA studies (3-fold increase at best relative to wild type (26)), mismatches of G–G and T–T that would lead to such transversion mutants are recognized and cut by mycobacteria NucS *in vivo* at essentially the same efficiency at G–T mismatches (this report). This discrepancy may be resolved by the supposition that G–T base pairs are by far the most highly generated mismatch generated *in vivo*, the repair of which has evolved to be quite efficient, and that G–G and T–T mismatches, while recognized and repaired by NucS, are simply not generated at the same high frequencies as G–T mismatches *in vivo*.

To unravel the mechanistic details of NucS MMR, we used oligo-mediated recombineering to examine exonucleolytic processing that takes place in the chromosome following the cutting of the chromosome at a repairable mismatch. This was done by delivering an oligo containing a G-G mismatch by recombineering and following the specificity and extension of an *in vivo* cut at that mismatch by measuring either loss or retainment of a nearby mismatch (C–T) as determined by the frequency of Hyg^R^ colonies following plating of the outgrowth. The assay, however, cannot distinguish between whether a nick or a dsDNA cut is made at the site of the mismatch, as this recombineering approach can only measure the activity of endogenous exonucleases on the strand containing the mispaired base supplied by the oligo (the top strand of the oligo highlighted in blue in Figure 6B). If a dsDNA cut were made by NucS, extensive processing of the *bottom* strand in Figure 6B would not lead to loss of Hyg^R^ transformants, as it would lead to digestion of the ‘T’ base of the C–T mismatch, leading to replacement of the stop codon in the defective *hyg* gene.

By comparing the number of bases needed to process the top strand in Figure 7A by digestion of either a 5′ end or a 3′ end, it is clear that the 5′ end is processed more efficiently than the 3′ end, as seen in Table 3. However, what is just as telling in this experiment is the limited nature of the processing of the 5′-3′ exonuclease following the cut made by NucS at a mismatch. By examining the extent of loss of Hyg^R^ transformants among the oligos that generate mismatches that confer Hyg^R^ (C–T) and a substrate for NucS cutting (G-G), oligos ‘–3’ to ‘–20’ in Figure 7B, one can observe that processing of the NucS-induced cut by a 5′-3′ exonuclease in most cases extends to 8 bases from the cut site (including the five base 5′ overhang). Digestion of an additional 3 bases (11 total) is diminished 20-fold, as evidenced by a 20-fold increase in the frequency of Hyg^R^ transformants seen with oligo -8 relative to -5 in Figure 7B. Ultimately, the use of an oligo where the distance between the C-T and G-G mismatches generated at the replication fork is 13 bases (oligo -13) results in no loss of Hyg^R^ transformants relative to the control oligo. These results suggest that digestion of the 5′-3′ strand from the site of a NucS cut (or nick) involves at least 8 base pairs, but not more than ∼12 base pairs. This result means that if NucS makes a dsDNA cut at a mismatch *in vivo*, it is not further processed by RecA-promoted homologous recombination events, as efficient recombination requires much larger sequences of homology (∼200 bases) relative to what is observed here (40). Limited processing of a NucS-induced cut *in vivo* was also recently observed by a deep sequencing methodology (41) using the same *M. smegmatis* strain constructed for this report (see companion paper).

The use of a double-mismatch generating oligo was also used to test the genetic dependency of NucS MMR. It is reasoned that if a NucS-promoted dsDNA cut is made *in vivo* at a mismatch, then inactivating mutations of any downstream functions involved in MMR would lead to non-repair of the dsDNA cut and subsequent death of the cell. In a similar vein, if only a nick is made at the mismatch by NucS, loss of any functions required for exonucleolytic processing of the nick would likely lead to some frequency of indel formation and thus to loss of Hyg^R^ transformants. Our oligo recombineering assay and subsequent sequencing of the region of *hyg* containing the G-G mismatch showed that neither RecA nor RadA is involved in downstream processing of the cut made by NucS, consistent with the limited processing of cuts made *in vivo* shown in Figure 7. We next examined if the non-homologous end-joining functions Ku and LigD might somehow be involved MMR in *M. smegmatis*. Since mycobacteria exhibit the uniqueness of possessing both the novel NucS MMR system and NHEJ functions (neither present in most bacteria), it was hypothesized that NHEJ, together with restriction enzyme-like NucS, might work together to repair mismatches following a NucS-promoted dsDNA cut, in a way the exhibits a high-fidelity version of NHEJ-promoted repair of dsDNA breaks. However, we found no effect on the frequency of Hyg^R^ transformants (or repair of the G–G mismatch by sequencing) with the double-mismatch generating oligo in a strain deleted of both Ku and LigD, showing that NHEJ functions are not involved in MMR in mycobacteria.

Finally, the limited processing of the cut made *in vivo* was highly suggestive of a flap endonuclease, where a cut is made at the ssDNA-dsDNA junction to remove a 5′ single-stranded DNA flap. In this scenario, one imagines that NucS cutting *in vivo* is limited to only the replicative strand (by some unknown function) and that unwinding from the nick is limited to the generation of a 5′ flap containing the mispaired base. Subsequent cutting of the flap by a resident endonuclease would excise a small ssDNA fragment containing the mispaired base. However, the deletion of the gene that encodes the FenA 5'-flap endonuclease (MSMEG_3883) had no effect on the frequency of Hyg^R^ transformants (or in the repair of the G-G mismatch) in our double-mismatch generating oligo assay, ruling out a role of *fenA* in NucS-promoted MMR, or that some other exonuclease *in vivo* can substitute for FenA in its absence. In these types of cases, it may require double (or multiple) exonucleolytic mutants to be generated to determine if processing of the NucS-induced break is carried out by FenA-like overlapping functions. Along this line, a double MSMEG_5004 (dsDNA exonuclease) *fenA* double mutant is being constructed to address this question.

One possibility for the function that supplies the 5′-3′ exonuclease activity in NucS-promoted MMR is Polymerase I (encoded by *polA*) as it possesses such an activity in its C-terminal domain (where its removal results in the generation of the Klenow fragment in *E. coli*) and is already at the replication fork in its role to trim RNA primers and fill-in the gaps between Okazaki fragments. However, testing this hypothesis with our recombineering assay would require a strain with a mutation in the C-terminal domain that inactivates its 5′-3′ exonuclease function. Unfortunately, removal of Pol I 5′-3′exonuclease lowers oligo recombineering rates 9-fold in *E. coli*,which could make such an assay to test for Pol I involvement in mycobacterial MMR challenging. Nonetheless, such a mutant is under construction.

Thus, downstream components of the NucS MMR pathway remain elusive as we continue to use this assay to examine the role of other nucleases and helicases known to exist in the mycobacterial genome that may have a role in high fidelity repair of mismatched bases in the wake of the replication fork. If in fact, the mycobacterial NucS protein makes a dsDNA break at the mismatch *in vitro* (like its *C. glutamicum* counterpart), the mechanism *in vivo* would have to rely on a modification of NucS activity *in vivo* (perhaps by binding of an interacting partner) such that nicking of the template strand is prevented. Alternatively, one can imagine that a dsDNA cut at the mismatch is made *in vivo* but is quickly held together via the action of an SMC-like protein (perhaps the *rad50* analog MSMEG_5590) that promotes a scaffold for quick religation of the leading strand that doesn’t expose the cell to the deleterious effects of a dsDNA break. Further strains containing deletions of such genes will be constructed and tested in our double-mismatched oligo assay.

## Supplementary Material

gkae895_Supplemental_File

## Data Availability

The data underlying this article, including plasmid maps and sequences, will be shared on reasonable request to the corresponding author. Plasmids pKM497 (PgroEL expressor), pIR540 (NucS-producer) and pKM585 (*attB* containing defective *hyg* gene) will be made available on the Addgene plasmid depository site *addgene.com*.
