# Supplementary material for: Mycobacterium smegmatis NucS-promoted DNA mismatch repair involves limited resection by a 5′-3′ exonuclease and is independent of homologous recombination and NHEJ
Source: Nucleic Acids Res. 2024 Oct 17;52(20):12308–23. doi: 10.1093/nar/gkae895 (PMC11551767; doi:10.1093/nar/gkae895)
Supplement: gkae895_Supplemental_File [file gkae895_supplemental_file.docx]

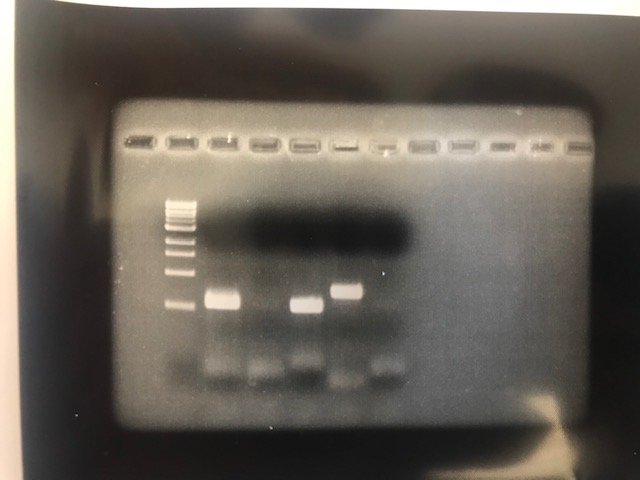

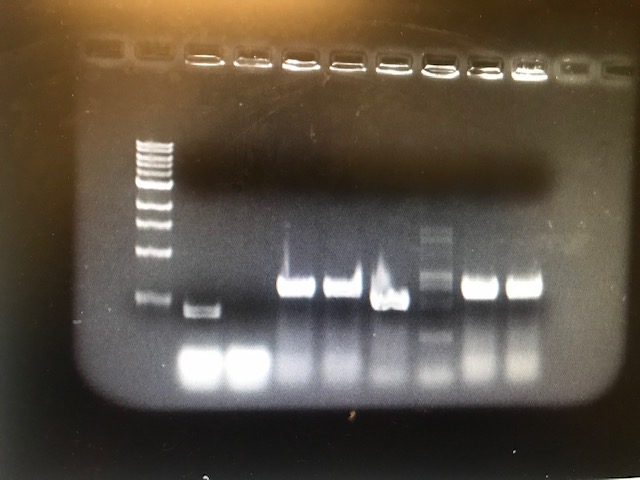



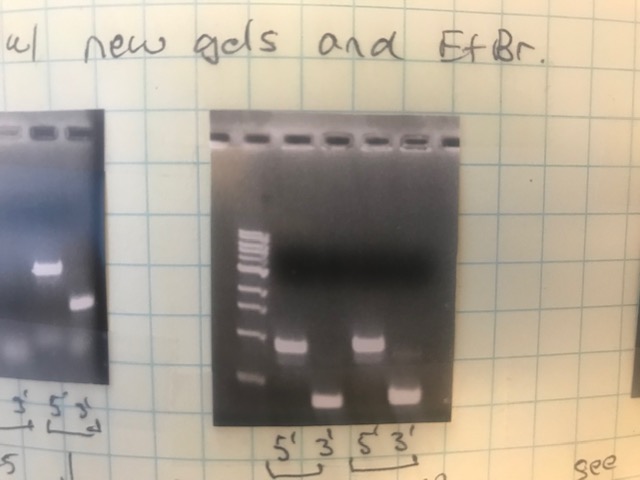

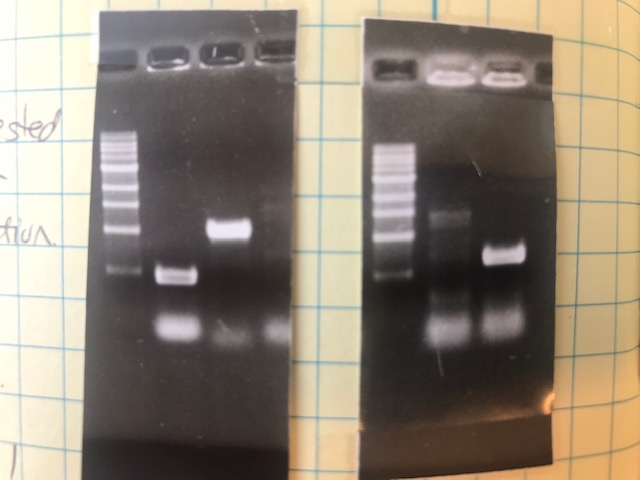

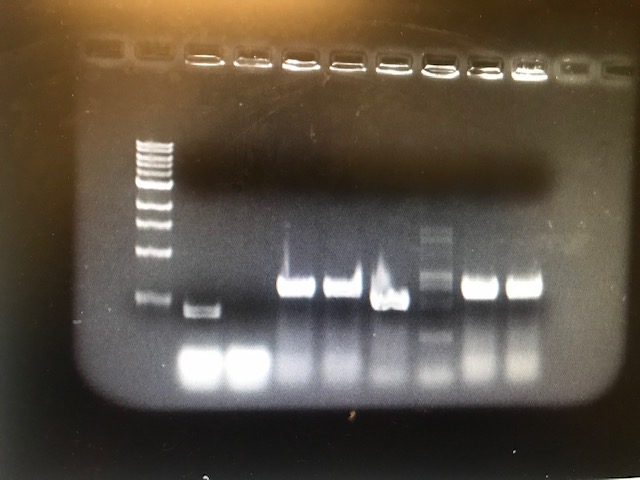

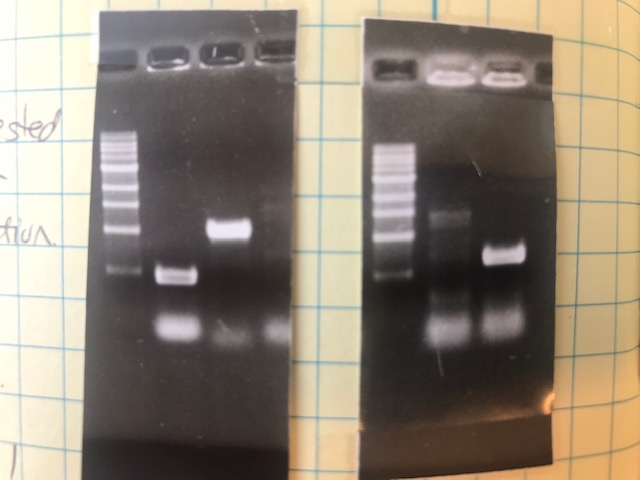


Fig. S1 – **PCR verifications of M. smegmatis KO mutants.**

**A-C.** Primers internal to *nucS*, *radA*, and *fenA* were used to verify that genes that were present in wild type cells were absent in the ORBIT-generated KO mutants. Primers upstream and downstream of the target genes were also used to verify the presence of 5’ and 3’ junctions of the ORBIT integration vectors and the chromosomes, as described in Murphy et al (2018). Primer sequences are listed in Table S2.

**D-E**. Internal primers were also used to verify the absence of *recA* and *Ku* genes from strains obtained from the Glickman lab. Control PCRs were performed to generate amplicons for internal regions of the *leuB* genes to show cells were competent for colony PCR. PCRs were done as described in Murphy et al (2018). Primer sequences are listed in Table S2.

Primers: *radA radA* 5’ junc 3’ junc

Train: WT Δ*radA*  Δ*radA* Δ*radA*

Figure S1

D.

E.

Primers: *Ku Ku leuB leuB*

Train: WT Δ*Ku* WT Δ*Ku*

Primers: *recA recA leuB leuB*

Train: WT Δ*recA*  WT Δ*recA*

Primers: *nucS nucS* 5’ junc 3’ junc

Strain: WT Δ*nucS.* Δ*nucS* Δ*nucS*

Primers: *fenA fenA* 5’ junc 3’ junc

Train: WT Δ*fenA* Δ*fenA*  Δ*fenA*

A.

B.

C.

Fig. S2A. **Plasmid map for pKM497.** The plasmid is a Giles integration vector for the cloning and expression of genes in *M. smegmatis* and *M. tuberculosis*. Plasmid (and sequence) available from addgene.com.

Figure S2A

**Plasmid map for pIR540.** Plasmid is a derivative of pKM497 for the expression of *nucS* in *M. smegmatis*. Plasmid (and sequence) available from addgene.com.

Figure S2B

**Plasmid map for pKM585.** Plasmid is an integration vector for the native Bxb1 *attB* site that carries a defective hyg gene. Integrase must be supplied in trans (*e.g.* pKM461) Plasmid (and sequence) available from addgene.com.

Figure S2C


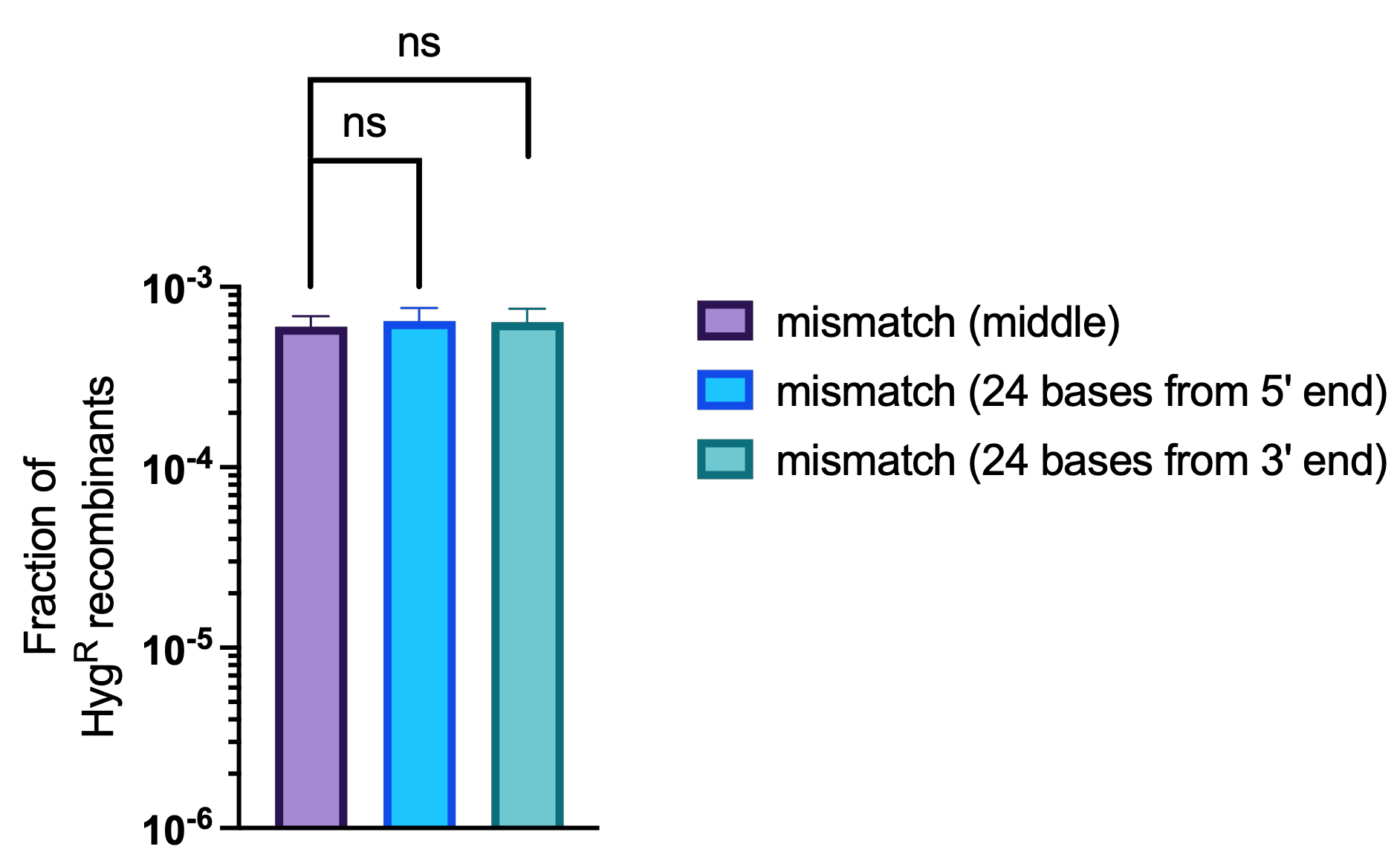


Figure S3

Fig. S3 –  **Oligo recombineering frequencies as a function of the position of base within the oligo that restores Hyg^R^.**

*M. smegmatis cells* containing pKM461 (RecT and Bxb Int) and pKM614 (Orbit integration vector containing the defective hyg gene) were electroporated with oligos where the base restoring Hyg^R^ was in the middle of the oligo (position 44 of an 87 mer) or placed 24 bases from either the 5’ or 3’ end of 87 mer oligos. Experiments were done in triplicate and SDs are shown. No differences in the frequencies of Hyg^R^ transformants were observed among these three oligos, signifying no significant loss of bases positioned at 24 bases from the ends of these oligos by endogenous nucleases after annealing to the lagging strand template at the replication fork. Oligos used were Hyg repair-CT, Hyg repair-CT-5’24, and Hyg repair-CT-3’24 (see Table S2 for sequences).


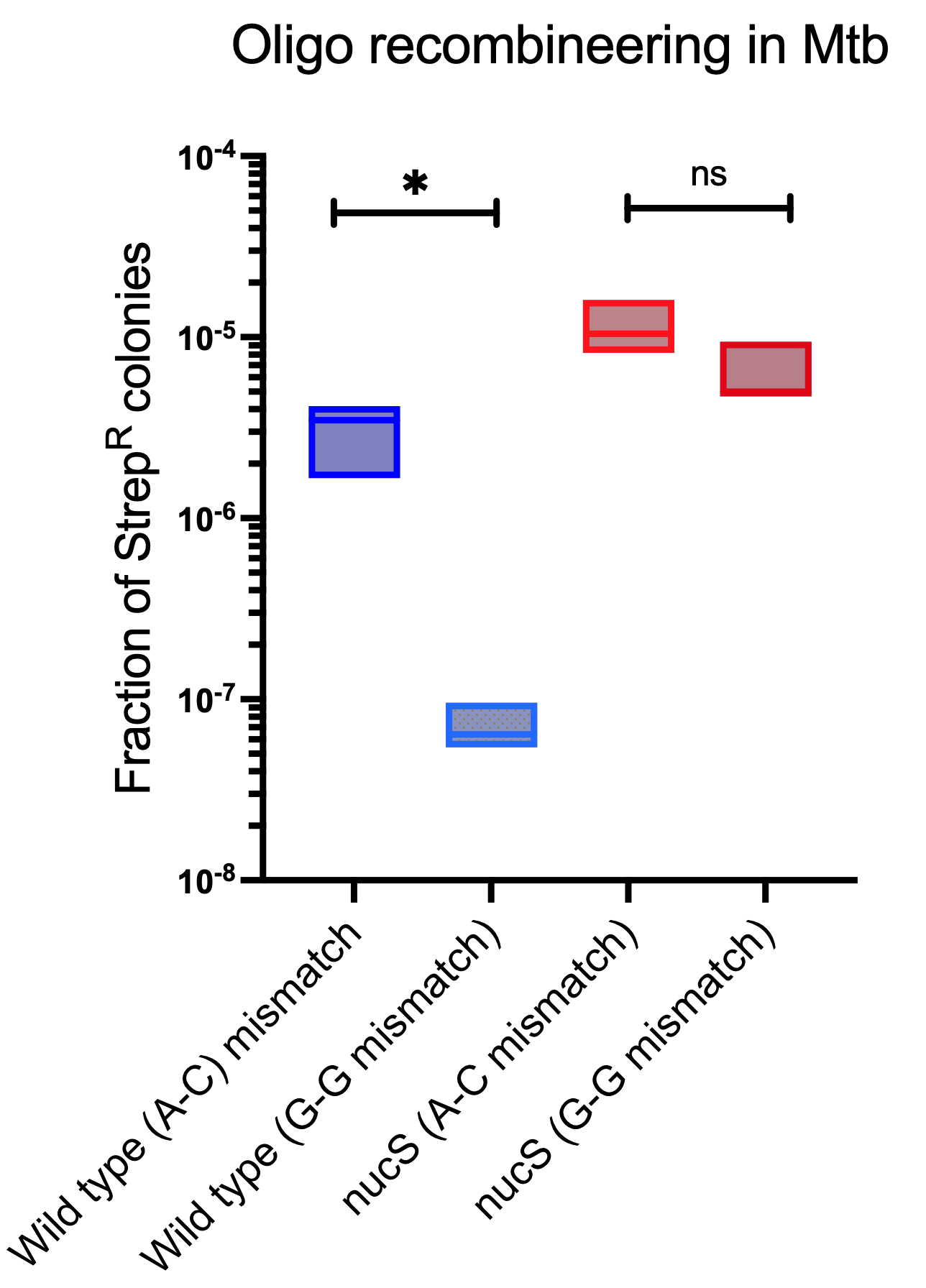


Figure S4

**Recombineering of the *rpsL* locus in *M. tuberculosis*.** An assay was set up like the one shown in Table 1 but testing MMR in *M. tuberculosis*. Both wild type (H37Rv) and a ΔnucS derivative (ΔRv3122) were electroporated with oligos targeting the *rpsL* gene, where either a predicted repairable mismatch (G-G) or unrepairable mismatch (A-C) was generated. In H37Rv cells, the A-C mismatch is left unrepaired leading to high levels of streptomycin resistance, whereas the G-G mismatch is repaired leading to low levels of streptomycin resistance. Both mismatches lead to high levels of streptomycin resistance in the ΔnucS derivative. Experiment was done in triplicate. Oligos used in this experiment are listed in Table S2: Mtb-rpsL-K43R (A-C mismatch) and Mtb-rpsL-K43N (G-G mismatch). This experiment was caried out in a BSL3 facility governed by the Institutional Biosafety Office at UMass Chan Medical School. *P < 0.05


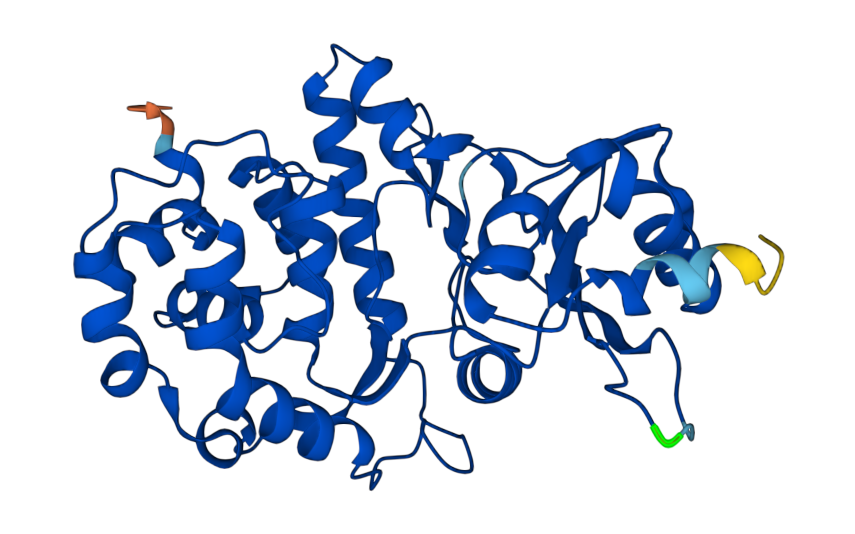


Figure S5

A.

Glycine-110

B.

| *Streptomyces hygroscopicus1* | GRGELRPG**TGA**WPWPYLV |
| --- | --- |
| *Streptomyces varsoviensis* | GRGELRPG**GAG**WPWPYLV |
| *Streptomyces griseocarneus* | **A**RGELRPGT**D**AWPWPFLV |
| *Streptomyces luteoverticillatus* | GRGEL**F**PG**GDG**WPWPYLV |
| *Saccharothrix australiensis* | G**A**GELRPG**SPT**WPWPFLV |
| *Streptoalloteichus hindustanu*  *Streptomyces hygroscopicus* | GRGELRP**SGPG**WPWP**YV**V  GRGELRPGT**R**AWPWPYLV |
|  |  |

Figure S5

**Description of the defective hygromycin phosphotransferase gene.** (A) In order to develop an assay where one could generate multiple types of mismatches at a stop codon placed within a selectable gene, which would also require the protein to still be active independent of the type of amino acid replacing the stop codon, we turned to the gene that confers hygromycin resistance (Hyg^R^). In the alpha fold structure of the hygromycin phosphotransferase gene, an extended loop at the surface of the protein was found to include a glycine at position 110. We thus replaced the glycine codon GAA with the TGA stop codon using recombineering. The assay ultimately revealed that all amino acid substitutions made at this position were tolerant.

(B) The capability of replacing glycine-110 in *hyg* with multiple types of amino acids was also favored following a search of the protein sequences from various hygromycin phosphotransferases in GenBank. Clearly, Gly-110 is not highly conserved.

Figure S6

Alignment of *M. smegmatis* NucS (224 aa) with *T. kodakarensis* NucS (252 aa)

**31.4% identity in 204 residues overlap; Score: 195.0; Gap frequency: 2.0%**

***M. smegmatis* 1 MRLVIAQCTVDYVGRLTAHLPSARRLLLFKADGSVSVHADDRAYKPLNWMSPPCWVTEQD**

***T. kodakarensis* 30 MLTIFARCKVHYDGRAKSELGSGDRVIIVKPDGSFLIHQSKKR-EPVNWQPPGSRVRLEL**

*** * * * * ** * * * * *** * * ** * ***

***M. smegmatis* 61 TETGVALWVVENKTGEQLRITVEDIEHDSHHELGVDPGLVKDGVEAHLQALLAEHVELLG**

***T. kodakarensis* 89 RENPV-LVSIRRKPRETLEVELEEVYMVSVFRAEDYEELALTGSEAEMAELIFENPEVIE**

*** * * * * * * * * * ** * * ***

***M. smegmatis* 121 AGYTLVRREYPTPIGPVDLLCRDELGRSVAVEIKRR-GEIDGVEQLTRYLELLNRDSLLA**

***T. kodakarensis* 148 PGFKPLFREKAIGTGIVDVLGRDSDGNIVVLELKRRRAELHAVRQLKSYVEIL-REEYGD**

*** ** * ** * ** * * * *** * * ** * * * ***

***M. smegmatis* 180 PVAGVFAAQQIKPQARTLATDRGI**

***T. kodakarensis* 207 KVRGILVAPSLTSGAKRLLEKEGL**

*** * * * * ***

# Figure S6 – NucS alignment - Alignment was done using the SIM Alignment Tool for Protein sequence (web.expasy.org). Residues suspected being important for NucS exonucleolytic activity are highlighted in red, whereas residues expected as being important for DNA binding are highlighted in green (see Nakae et al, Ref. 14).

Table S1

Hyg repair oligos used in the mismatch specificity assay

|  | Hyg repair oligos | |  |  |  | mismatch | new codon | Amino acid |
| --- | --- | --- | --- | --- | --- | --- | --- | --- |
| Wild type | 5’ ATCCGGCTCATCACCAGGTAGGGCCACGGCCAGGC**TCC**GGTGCCGGGCCGCAGCTCGCCGCGGCCGAGGA 3’ | | | | |  |  |  |
| Stop codon | 5’ ATCCGGCTCATCACCAGGTAGGGCCACGGCCAGGC**CTA**GTTGCCGGGCCGCAGCTCGCCGCGGCCGAGGA 3’ | | | | |  |  |  |
| Oligo # | Position (1) 110 GLYCINE G | |  |  |  |  |  |  |
| 1 | 5’ ATCCGGCTCATCACCAGGTAGGGCCACGGCCAGGC**TCC**GGTGCCGGGCCGCAGCTCGCCGCGGCCGAGGA 3’ | | | | | multiple | GGA | glycine |
| 2 | 5’ ATCCGGCTCATCACCAGGTAGGGCCACGGCCAGGC**CTT**GGTGCCGGGCCGCAGCTCGCCGCGGCCGAGGA 3’ | | | | | T/T | AAG | Lysine |
| 3 | 5’ ATCCGGCTCATCACCAGGTAGGGCCACGGCCAGGC**CTG**GGTGCCGGGCCGCAGCTCGCCGCGGCCGAGGA 3’ | | | | | G/T | CAG | Glutamine |
| 4 | 5’ ATCCGGCTCATCACCAGGTAGGGCCACGGCCAGGC**CTC**GGTGCCGGGCCGCAGCTCGCCGCGGCCGAGGA 3’ | | | | | C/T | GAG | Glutamic Acid |
|  | Position (2) 110 GLYCINE G | |  |  |  |  |  |  |
| 5 | 5’ ATCCGGCTCATCACCAGGTAGGGCCACGGCCAGGC**CGA**GGTGCCGGGCCGCAGCTCGCCGCGGCCGAGGA 3’ | | | | | G/A | TCG | Serine |
| 6 | 5’ ATCCGGCTCATCACCAGGTAGGGCCACGGCCAGGC**CCA**GGTGCCGGGCCGCAGCTCGCCGCGGCCGAGGA 3’ | | | | | C/A | TGG | Tryptophan |
| 7 | 5’ ATCCGGCTCATCACCAGGTAGGGCCACGGCCAGGC**CAA**GGTGCCGGGCCGCAGCTCGCCGCGGCCGAGGA 3’ | | | | | A/A | TTG | Leucine |
|  | Position (3) 110 GLYCINE G | |  |  |  |  |  |  |
| 8 | 5’ ATCCGGCTCATCACCAGGTAGGGCCACGGCCAGGC**GTA**GGTGCCGGGCCGCAGCTCGCCGCGGCCGAGGA 3’ | | | | | G/G | TAC | Tyrosine |
| 9 | 5’ ATCCGGCTCATCACCAGGTAGGGCCACGGCCAGGC**ATA**GGTGCCGGGCCGCAGCTCGCCGCGGCCGAGGA 3’ | | | | | A/G | TAT | Tyrosine |
|  |  |  |  |  |  |  |  |  |
| The top two rows show the reverse complement of a central region of the *hyg* gene, with the position of the Gly codon and the stop codon in bold face. |  | | | | |  |  |  |

Table S2

Oligonucleotides used for ORBIT-generated mutations and

Oligo-mediated recombineering

Identifier (Gene) ORBIT integration plasmid

**ORBIT:**

ΔMSMEG_4923 (ΔnucS) pKM464

5’TCAGAAGAGCCGGTACTCGTCGCTGTCCATTCCGCGCATCTGGTCGTAATCGAGTGTCACACAACGGATTGGTTTGTACCGTACACCACTGAGACCGCGGTGGTTGACCAGACAAACCGGGCAGAGGGCAGATGAGCGGTGAGCCGGCCGACGTAGTCGACGGTGCACTGGGCTATCACGAGGCGCAC 3’

ΔRv1321 (ΔnucS) pKM488

5’TTTGCGGCGCCGAGCCATCGCATCAGTTTAATCGCGCAACTCAGAACAGCCGGTACTCGCCGCTATCCATGGTTTGTACCGTACACCACTGAGACCGCGGTGGTTGACCAGACAAACCCTGGGCGATGACTAGACGCACCCGACTCACCTTAGAGCGCGCAACGACGTTGTTCCTTAGAGCGTGACCG3’

ΔMSMEG_6079 (ΔradA) (pKM611)

5’CGCAGGCCCGGCTGTCGGTGCCTGCCGATACTGTCACGGCGTGGCCGGTTCGAAAATACGTTCGCAGTACGGTTTGTCTGGTCAACCACCGCGGTCTCAGTGGTGTACGGTACAAACCCGGGAGATCGCGATTGCGGGTGCCCAATAGGATCGGGACAATACCGGCCGATCCTAGGAGGGCTGATGGC3’

ΔMSMEG_3883 (ΔfenA) (pKM611)

5’GTCATGATTCACGATACGTGGGACCGCGACTGTCCCTGCCGTGGTGTGTGACATCGACGTCGAGGTAGCCGGTTTGTCTGGTCAACCACCGCGGTCTCAGTGGTGTACGGTACAAACCCAGACCGCACTGGACCAGCTGCCCGACTGAGCCGGTCTACTTCGGCCGGCCGACCTCGTAGGTGCCGTCG3’

MSMEG_1397- rpsL intergenic region insertion (pKM614)

5’CACCACAATACCAGGGCCGACCGCGACGAAACAAACTCGTGCACGGCGGTCTAATCGCAGGTCAGACAGTGGTTTGTACCGTACACCACTGAGACCGCGGTGGTTGACCAGACAAACCGAGGTCCACCCTACGCTTCGCGACGCTCCATCCCGGAGGCCAGGCGCAGCAGCATATCGGTGAAAACCGC3’

**Oligo-Recombineering:**

Smeg-rpsL-K43R (base substitution; 70 mer)

5’ CGCGCGCGACCTTCCGGAGCGCCGAGTTCGGCTTC**c**TCGGAGTGGTGGTGTAAACGCGCGTGCACACGCC 3’

Smeg-rpsL-K43N (base substitution; 70 mer)

5’ CGCGCGCGACCTTCCGGAGCGCCGAGTTCGGCTT**g**TTCGGAGTGGTGGTGTAAACGCGCGTGCACACGCC 3’

Mtb-rpsL-K43R (base substitution; 70 mer)

5’ CGCGGGCAACCTTCCGAAGCGCCGAGTTCGGCTTC**c**TCGGAGTGGTGGTGTACACGCGGGTGCATACACC 3’

Mtb-rpsL-K43N (base substitution; 70 mer))

5’ CGCGGGCAACCTTCCGAAGCGCCGAGTTCGGCTT**g**TTCGGAGTGGTGGTGTACACGCGGGTGCATACACC 3’

Smeg-leuB-R101+1 (one bp insertion:74 mer)

5’ AGCGGGCCCGTCACACCCGGATACAGCCTGCCGGGGCG**g**CAGATTCACGTGATGGTCGAGCGCGAACCGCAGTT 3’

Smeg-leuB-R101Δ1 (one bp deletion:72 mer)

5’ AGCGGGCCCGTCACACCCGGATACAGCCTGCCGGGG**CC**AGATTCACGTGATGGTCGAGCGCGAACCGCAGTT 3’

Smeg-leuB-R101Δ2 (two bp deletion:71 mer)

5’ AGCGGGCCCGTCACACCCGGATACAGCCTGCCGGGG**CA**GATTCACGTGATGGTCGAGCGCGAACCGCAGTT 3’

Smeg-leuB-WT (73 mer)

5’ AGCGGGCCCGTCACACCCGGATACAGCCTGCCGGGGCGCAGATTCACGTGATGGTCGAGCGCGAACCGCAGTT 3’

Smeg-NucS-D138A (base substitution; 73 mer)

5’ GCGACCGAACGGCCCAGCTCGTCGCGGCACAACAGG**g**CGACGGGCCCGATCGGGGTCGGATACTCGCGGCGCA 3’

Smeg-NucS-E152A (base substitution; 73 mer)

5’ TGTTCGACGCCGTCGATCTCGCCGCGGCGCTTGATC**g**CCACGGCGACCGAACGGCCCAGCTCGTCGCGGCACA 3’

Smeg-NucS-K154A (base substitutions; 73 mer)

5’ GTCAGCTGTTCGACGCCGTCGATCTCGCCGCGGCGC**gc**GATCTCCACGGCGACCGAACGGCCCAGCTCGTCGC 3’

Smeg-NucS-Y12A (base substitutions; 73 mer)

5’ CGGGCAGAGGGCAGATGAGCGGTGAGCCGGCCGACG**gc**GTCGACGGTGCACTGGGCTATCACGAGGCGCACCC 3’

Smeg-NucS-N48G (base substitutions; 73 mer)

5’ TCCTGTTCGGTGACCCAGCACGGCGGGCTCATCCAG**gc**CAACGGCTTGTAGGCGCGGTCGTCGGCGTGCACGC 3’

Smeg-NucS-W49G (base substitutions; 73 mer)

5’ GTGTCCTGTTCGGTGACCCAGCACGGCGGGCTCATagcaTTCAACGGCTTGTAGGCGCGGTCGTCGGCGTGCA 3’

Hyg repair-32 (base substitutions; 84 mer)

5’GACGCCCCGGTGCCGGTGCCCCGCCT**t**CTCGGCCGCGGCGAGCTGCGGCCCGGCACCT**c**GGCCTGGCCGTGGCCCTACCTGGTG 3’

Hyg repair-CT (base substitution; 87 mer)

5’GTGCCCCGCCTCCTCGGCCGCGGCGAGCTGCGGCCCGGCACCT**c**GGCCTGGCCGTGGCCCTACCTGGTGATGAGCCGGATGACCGGC 3’

Hyg repair-CT-5’24 (base substitution; 87mer)

CGGCGAGCTGCGGCCCGGCACCT**c**GGCCTGGCCGTGGCCCTACCTGGTGATGAGCCGGATGACCGGCACCACCTGGCGGTCCGCAAT

Hyg repair-CT-3’24 (base substitution; 87mer)

TGGCGGACGCCCCGGTGCCGGTGCCCCGCCTCCTCGGCCGCGGCGAGCTGCGGCCCGGCACCT**c**GGCCTGGCCGTGGCCCTACCTGG

Base pair changes or additions are highlighted in red in lower case.

Positions of base pair deletions are highlighted in red and underlined.

**Gene knockout verifications**: (U-upstream; D-downstream; F-internal forward; R-internal reverse)

Smeg nucS-U

5’ CTGTTCTGCCGTGTGACGGTTTCC 3’

Smeg nucS-D

5’ GTCTGCTGTACGACGCACCCAAGC 3’

Smeg nucS-F

5’ TGTTCGCCGCGCAGCAGATCAAAC 3’

Smeg-nucS-D2

5’ CCTGTCGCACCTCGTAGTCGTAGC 3’

Smeg-radA-U

5’ TCCAGCGAACACTAGGTCTTCACC 3’

Smeg-radA-D

5’ TGCACGACGTTGCGTCCGGATCTC3’

Smeg-radA-F

5’ ATCAGGTGTACCTGGCCGCCGAAT 3’

Smeg-radA-R

5’ TTGCTGTTGATCGGCATGCGGCAG

Smeg-fenA-U

5’ CGGACGTAGAAATCAACGGTGGCC 3’

Smeg-fenA-D

5’ GACACCTTCACCTGCGAGGTGAGC 3’

Smeg-fenA-F

5’ ACATCGAGGAGGTTCCCGACGACC 3’

Smeg-fenA-R

5’ GGTGCGATGTGCCTTGGACAGACC 3’

Smeg-recA-U

5’ ACGGTAGGCCTGTCGTGTGCCGTC 3’

Smeg-recA-R

5’ CTCCGGGCCGTAGATCTCGATGAC 3’

Smeg-Ku-F

5’ TCAACAAGGCCTTCGAATCCGACG 3’

Smeg-Ku-R

5’ CTGATAGTCGTCGTGGTACAGGTC 3’
